# Asian continental isoscapes of tree-ring δ^18^O: implications for paleoclimate and paleoenvironment

**DOI:** 10.1093/nsr/nwaf481

**Published:** 2025-11-07

**Authors:** Ru Huang, Chenxi Xu, Wenling An, Qingyu Zhao, Yaru Zhao, Yucheng Liu, Jussi Grießinger, Wolfgang Jens-Henrik Meier, Zhengtang Guo

**Affiliations:** State Key Laboratory of Lithospheric and Environmental Coevolution, Institute of Geology and Geophysics, Chinese Academy of Sciences, Beijing 100029, China; Department of Environment and Biodiversity, University of Salzburg, Salzburg 5020, Austria; State Key Laboratory of Lithospheric and Environmental Coevolution, Institute of Geology and Geophysics, Chinese Academy of Sciences, Beijing 100029, China; College of Earth and Planetary Sciences, University of Chinese Academy of Sciences, Beijing 100049, China; State Key Laboratory of Lithospheric and Environmental Coevolution, Institute of Geology and Geophysics, Chinese Academy of Sciences, Beijing 100029, China; State Key Laboratory of Lithospheric and Environmental Coevolution, Institute of Geology and Geophysics, Chinese Academy of Sciences, Beijing 100029, China; State Key Laboratory of Lithospheric and Environmental Coevolution, Institute of Geology and Geophysics, Chinese Academy of Sciences, Beijing 100029, China; State Key Laboratory of Lithospheric and Environmental Coevolution, Institute of Geology and Geophysics, Chinese Academy of Sciences, Beijing 100029, China; College of Earth and Planetary Sciences, University of Chinese Academy of Sciences, Beijing 100049, China; Department of Environment and Biodiversity, University of Salzburg, Salzburg 5020, Austria; Department of Environment and Biodiversity, University of Salzburg, Salzburg 5020, Austria; State Key Laboratory of Lithospheric and Environmental Coevolution, Institute of Geology and Geophysics, Chinese Academy of Sciences, Beijing 100029, China; College of Earth and Planetary Sciences, University of Chinese Academy of Sciences, Beijing 100049, China

**Keywords:** tree-ring oxygen isotopes, Asia, isoscapes, atmospheric circulation, paleoclimate, paleoenvironment

## Abstract

Continental-scale isoscapes of tree-ring oxygen isotopes (δ^18^O_TR_) are crucial for understanding atmospheric circulation dynamics, interpreting climatic significance, and tracing wood provenance. However, continental-scale δ^18^O_TR_ isoscapes remain underdeveloped. We compiled 313 multi-year averaged δ^18^O_TR_ records across Asia and generated isoscapes using two machine learning approaches: XGBoost and Random Forest. Results reveal a ‘sandwich’ pattern: depleted values at high (>50°N) and low (<30°N) latitudes, enriched values at mid-latitudes (30°N–50°N). This pattern closely resembles the distribution of precipitation δ^18^O (δ^18^O_P_). Correlation and commonality analyses confirm δ^18^O_P_ as the primary driver of δ^18^O_TR_ isoscape patterns across Asia. Continental-scale δ^18^O_TR_–elevation relationships are generally insignificant, except in Indian Summer Monsoon regions showing significant negative correlations (*r* = −0.69, *p* < 0.05). These findings suggest that δ^18^O-based paleoaltimetry reconstructions work best in regions with dominant moisture sources such as Indian Summer Monsoon regions. This study provides Asia’s first continental-scale δ^18^O_TR_ isoscapes, establishing a foundation for atmospheric circulation and dendroprovenancing research.

## Teaser

By complying 313 multi-year averaged tree-ring oxygen isotope records across Asia and generated isoscapes using thin-plate spline regression interpolation, this study provides Asia's first continental-scale tree-ring dδ^18^O isoscapes, establishing a foundation for atmospheric circulation and dendroprovenancing research.

## INTRODUCTION

The term isoscapes was introduced in 2005 to describe spatially explicit models of isotopic variation across geographic regions [1]. Among these, isoscapes of precipitation oxygen isotopes (δ^18^O_P_) have become particularly valuable tools for understanding atmospheric circulation, hydrological processes, and both past and recent climate variability [1,2]. Significant progress has been made in constructing δ^18^O_P_ isoscapes on global [3] and regional scales [4]. Techniques such as spatial interpolation, dynamical modeling, and machine learning have produced increasingly detailed products [1,5]. However, the limited and sparse distribution of observational stations—particularly in remote and mountainous areas—continue to constrain the spatial resolution and completeness of δ^18^O_P_ isoscapes. This limitation underscores the importance of incorporating complementary proxy records, such as tree rings, to enhance isoscape reconstructions across broader spatial scales.

Tree-ring oxygen isotopes (δ^18^O_TR_) have demonstrated their strong potential as proxies for extending δ^18^O_P_ isoscapes. δ^18^O_TR_ is influenced by both the δ^18^O_P_ and relative humidity (RH) [6,7]. Through mechanistic models and transfer functions, δ^18^O_TR_ has shown promising capacity for reconstructing δ^18^O_P_ [8]. Advances in pre-treatment techniques and mass spectrometry have broadened δ^18^O_TR_ applications across a wide range of timescales [9], from intra-annual resolution [8,10] to millennial-scale hydroclimate reconstructions [11,12]. Spatially, δ^18^O_TR_ studies now encompass regions from the low-latitude tropics to mid- and high-latitude environments [13,14]. These methodological and spatial advances therefore provide a key foundation for developing large-scale δ^18^O_TR_ isoscapes.

Regional δ^18^O_TR_ isoscapes have been used to investigate atmospheric moisture transport, climate variability, and wood provenance. For example, δ^18^O_TR_ isoscapes (a network of δ^18^O_TR_) have identified the Indian Summer Monsoon (ISM) as the dominant moisture source for Southwest China [15]. Meanwhile, a network of 24 δ^18^O_TR_ chronologies across Asia has been used to decipher the combined influence of South Asia Summer monsoon precipitation and El Nino Southern Oscillation (ENSO) on regional δ^18^O_TR_ variability [16]. In North America, δ^18^O_TR_ isoscapes have revealed a decreasing influence of the Labrador Sea on forest moisture regimes [13]. Additionally, δ^18^O_TR_ isoscapes have proved to be helpful in wood provenance assessments, such as identifying timber origins in North America [17], Europe [18], tropical Africa [19] and tropical South America [19]. Despite these promising applications, continental-scale δ^18^O_TR_ isoscapes remain underdeveloped, largely due to the limited data availability.

Asia represents an ideal setting for advancing δ^18^O_TR_ isoscape research due to its diverse atmospheric circulation regimes, complex topography, and a relatively dense distribution of δ^18^O_TR_ records [9]. This study establishes the first δ^18^O_TR_ isoscape for the Asian continent, constructed from multi-year mean values compiled in previous work [9] and generated using two machine learning approaches—XGBoost and Random Forest—as described in earlier studies [2,5]. Our objectives for the large-scale δ^18^O_TR_ isoscape investigation were: (i) to characterize the spatial distribution of δ^18^O_TR_ across Asia; (ii) to identify environmental variables that mostly influence the isotopic patterns; and (iii) to quantify the relationship between δ^18^O_TR_ and elevation. To conduct this investigation, we propose two hypotheses. First, the spatial distribution of δ^18^O_TR_ is expected to reflect that of precipitation δ^18^O_P_, capturing the isotopic signature of source water incorporated during tree growth. Second, we anticipate that the relationships between δ^18^O_TR_ and environmental variables—such as δ^18^O_P_, RH, and elevation—will vary regionally, shaped by Asia’s climatic diversity and topographical complexity.

## RESULTS AND DISCUSSION

### Observed δ^18^O_TR_ isoscapes

**Figure 1.**
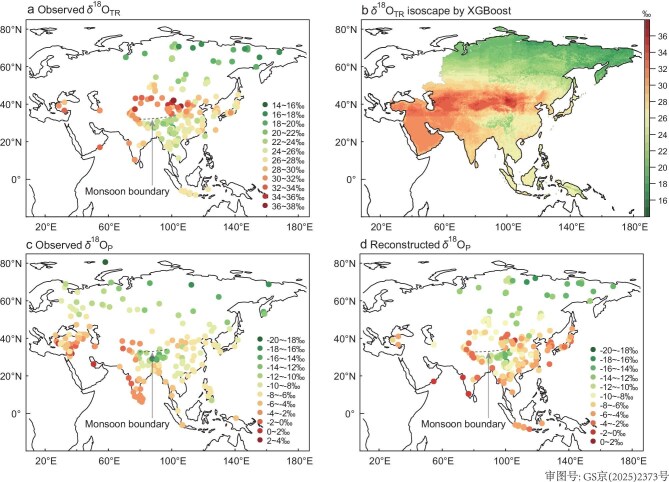
Observed and modelled spatial patterns of δ^18^O_TR_ and δ^18^O_P_ across Asia. (a) Observed δ^18^O_TR_ (313 sites). (b) δ^18^O_TR_ isoscape based on XGBoost. (c) May–September δ^18^O_P_ (252 sites) from the GNIP and TNIP [20]. (d) Reconstructed May–September δ^18^O_P_ derived from δ^18^O_TR_ using CRU TS 4.07 (1993–2022) RH data [21]. Black dotted lines in (a), (c) and (d) delineate the boundary of the ISM domain.

The observed δ^18^O_TR_ values ranged from 15.20‰ to 37.10‰ (Fig. 1a and b). Observed May–September δ^18^O_P_ from GNIP and TNIP stations varied between −18.05‰ and 2.93‰ (Fig. 1c). Reconstructed May–September δ^18^O_P_, derived from δ^18^O_TR_ and CRU RH data, ranged from −18.92‰ to 1.17‰ (Fig. 1d). Similarly, δ^18^O_P_ reconstructed using CHELSA RH varied from −8.84‰ to −0.60‰, with a mean of −9.07‰ and a median of −8.53‰ (Fig. S1). The mean difference between δ^18^O_TR_ and δ^18^O_P_ reflects several fractionation processes occurring after precipitation infiltrates the soil and during water transport and assimilation in trees [6]. While root uptake does not fractionate oxygen isotopes, leaf water becomes enriched through transpiration, and this signal is incorporated into cellulose during synthesis [6]. These processes collectively result in δ^18^O_TR_ values being higher than δ^18^O_P_.

The Asian δ^18^O_TR_ isoscapes exhibit a characteristic ‘sandwich’ pattern (Fig. 1a and b), characterized by more depleted values at high latitudes (>50°N) and low latitudes (<30°N), and relatively enriched values at mid-latitudes (30°N–50°N). This spatial configuration closely mirrors the observed May–September δ^18^O_P_ distributions from the GNIP and TNIP (Fig. 1c), thereby supporting our first hypothesis. The δ^18^O_P_ values reconstructed from observed δ^18^O_TR_ isoscapes and CRU RH data also replicate this ‘sandwich’ pattern (Fig. 1d). A similar structure was obtained using RH from the CHELSA dataset for δ^18^O_P_, reinforcing the robustness of these results (Fig. S1). These spatial patterns suggest a distinct climate transition zone, where we identify a monsoon boundary at approximately 31°N–33°N on the central Tibetan Plateau, which is consistent with previously reported boundaries based on extensive δ^18^O_P_ observations (34°N–35°N) [20]. Notably, the southeastern Tibetan Plateau shows the lowest δ^18^O_TR_ values due to isotopically depleted rainfall from its exceptionally wet upstream regions (e.g., Cherrapunji) [9]. These findings demonstrate the potential of δ^18^O_TR_ isoscapes in capturing large-scale isotopic gradients and atmospheric circulation patterns including dynamics of the Indian and East Asian monsoon systems.

### Simulated δ^18^O_TR_ isoscapes

**Figure 2.**
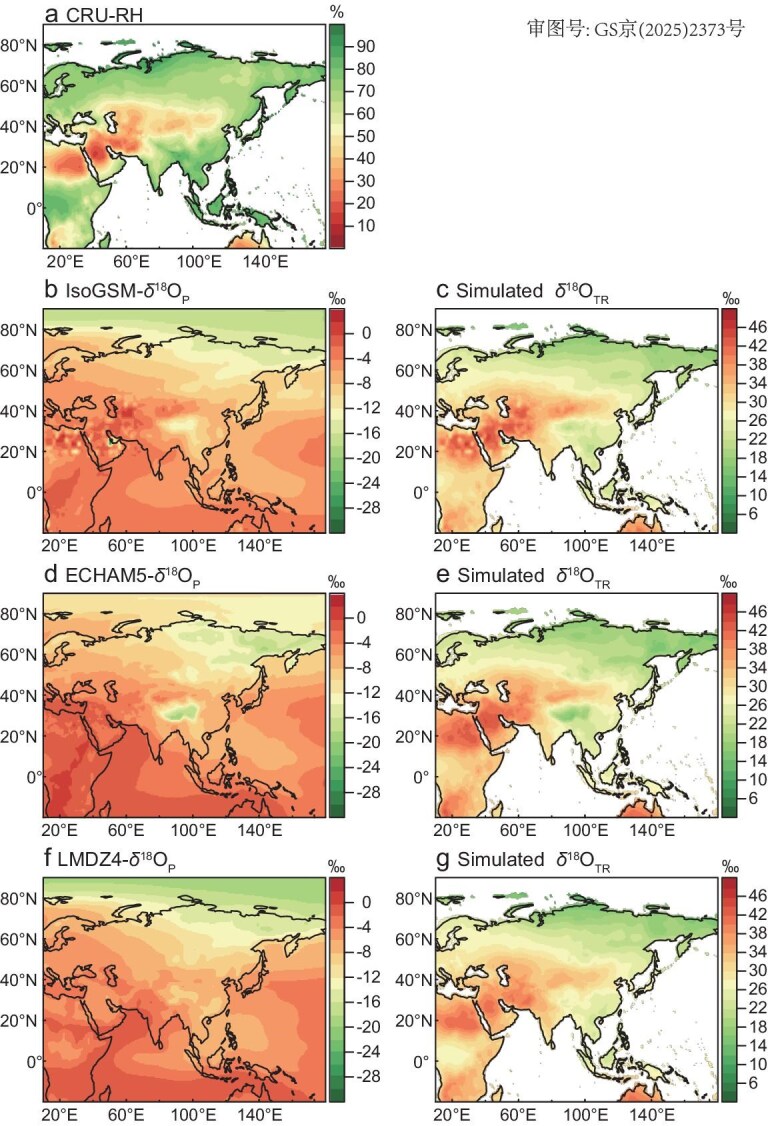
Spatial distribution of RH and simulated δ^18^O in precipitation and tree rings across Asia. (a) Mean May–September RH derived from the CRU dataset. (b), (d) and (f) Simulated May–September δ^18^O_P_ for the same period from three global isotope-enabled models: IsoGSM (b), ECHAM5 (d) and LMDZ4 (f). (c), (e) and (g) Corresponding simulations of δ^18^O_TR_ derived from each model, respectively.

We modeled δ^18^O_TR_ isoscapes using RH data derived from the CRU TS 4.07 dataset, along with simulated May–September δ^18^O_p_ values from multiple global isotope-enabled climate models (isoGSM, ECHAM5 and LMDZ4). As anticipated, the modeled δ^18^O_TR_ isoscapes also successfully reproduced the characteristic ‘sandwich’ pattern observed in empirical δ^18^O_TR_ measurements (Fig. 2c, e and g vs. Fig. 1b). These modeled δ^18^O_TR_ distributions also resembled the spatial patterns generated by δ^18^O_P_ simulations (Fig. 2b, d, f), which exhibit depleted values in high-latitude regions and low-latitude monsoon zones. These highlight the coherence between δ^18^O_P_ and δ^18^O_TR_. Similar results were observed when using CHELSA RH and simulated May–September δ^18^O_P_ (Fig. S2).

### Spatial drivers of δ^18^O_TR_: δ^18^O_P_ vs. RH

**Figure 3.**
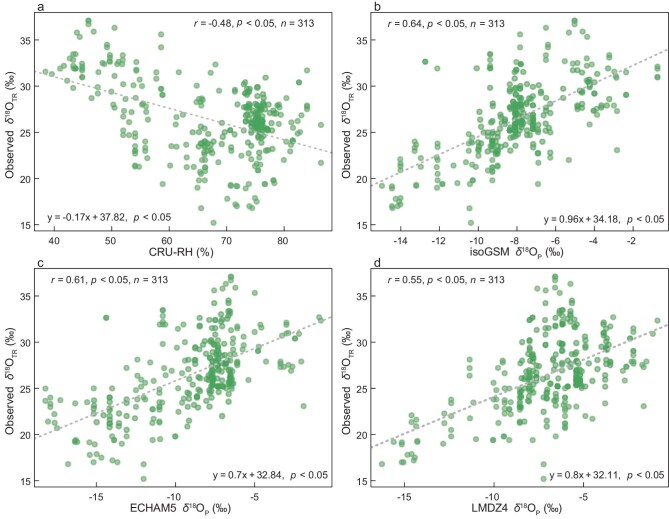
Relationships between observed δ^18^O_TR_ and RH/δ^18^O_P_ across 313 sampling sites in Asia. (a) Scatter plot illustrating the correlation between observed δ^18^O_TR_ values and mean May–September RH based on the CRU dataset. (b–d) Relationships between δ^18^O_TR_ and simulated δ^18^O_P_ from three global isotope-enabled models: IsoGSM (b), ECHAM5 (c) and LMDZ4 (d). Pearson correlation coefficients are shown at the top.

Multiple analytical approaches, including correlation analysis and commonality analysis, further demonstrate the predominant influence of δ^18^O_P_ in determining δ^18^O_TR_ isoscapes. The scatter plot reveals a negative relationship between RH and δ^18^O_TR_, with higher RH values corresponding to depleted δ^18^O_TR_ values (Fig. 3a). In contrast, we observe significant positive relationships between δ^18^O_P_ and δ^18^O_TR_ across all isotope-enabled models, indicating that enriched δ^18^O_P_ values correspond to enriched δ^18^O_TR_ values (Fig. 3b–d). Quantitatively, correlations between observed δ^18^O_TR_ and simulated δ^18^O_P_ (r = 0.55–0.64, *p* < 0.01, *n* = 313) are consistently stronger than those with RH (CRU: r = −0.48, *p* < 0.05; CHELSA: r = −0.47, *p* < 0.05; *n* = 313, Fig. 3a and Fig. S3). A commonality analysis (model: δ^18^O_TR_ = CRU RH + δ^18^O_P_) reveals that the combined effects of δ^18^O_P_ and RH explain 54.80%–62.73% of observed δ^18^O_TR_ variance (Table 1). Notably, the individual effect of δ^18^O_P_ (33.04%–39.81%) substantially exceeds that of RH (15.53%–25.86%), further confirming the dominant role of δ^18^O_P_ in determining δ^18^O_TR_ patterns across Asia. Similar results were obtained when using CHELSA RH data in the commonality analysis model (Table S1).

**Table 1.** Commonality analysis results showing the unique and shared contributions of CRU-RH and simulated δ^18^O_P_ to the spatial variability of δ^18^O_TR_ across Asia.

Model names of δ^18^O_p_	Individual effect of RH on δ^18^O_TR_ (%)	Individual effect of δ^18^O_p_ on δ^18^O_TR_ (%)	Joint effect on δ^18^O_TR_ (%)	Total effect on δ^18^O_TR_ (%)
IsoGSM	15.53	33.04	7.39	55.96
ECHAM5	25.86	39.81	−2.94	62.73
LMDZ4	24.57	31.88	−1.65	54.80

The dominant role of δ^18^O_P_ on δ^18^O_TR_ has also been observed in other regions such as eastern Canada [13], Eurasia [22], the Amazon [23] and the Europe Alps [24], although we acknowledge that the specific climatic regimes differ from those in Asia. The δ^18^O_P_ signal is first incorporated into plant water through soil moisture, and is subsequently transferred into photosynthetic products before ultimately being recorded in cellulose during synthesis [6].

### δ^18^O_TR_–elevation relationships

**Figure 4.**
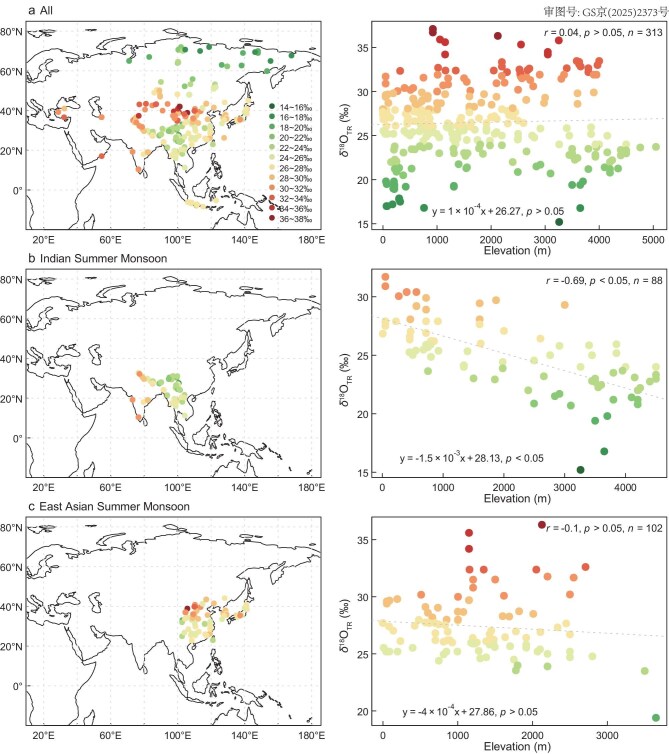
Relationship between δ^18^O_TR_ and elevation across Asia and regional monsoon domains. (a) Relationship between δ^18^O_TR_ and elevation for all 313 sites. (b and c) Same input data as (a) but for regional patterns within the ISM and EASM zones, respectively. For each panel, the left sub-figure displays the spatial distribution of sampling sites, and the right sub-figure shows the corresponding scatter plots of δ^18^O_TR_ vs. elevation. Pearson correlation coefficients are shown in the top right corner of the scatter plot. The definitions of each subregion are presented in Fig. S4.

Our second hypothesis, concerning the relationship between δ^18^O_TR_ and elevation, is also supported. The relationship between δ^18^O_TR_ and elevation demonstrates remarkable heterogeneity across the Asian continent, with distinctive patterns emerging at regional scales. No significant correlation between δ^18^O_TR_ and elevation is observed at continental scale (Fig. 4a, *r* = 0.04, *p* > 0.05). This lack of a continental-scale trend likely reflects the continent’s highly complex topography, the diversity of climate regimes, and the influence of multiple moisture sources—including local evapotranspiration, as well as long-distance transport from the Indian, Pacific and Atlantic Oceans [20,25].

In contrast, a significant negative δ^18^O_TR_–elevation relationship (Fig. 4b, *r* = –0.69, *p* < 0.05) is found in the ISM region, with a lapse rate of −0.15‰/100 m. This observation aligns with previous studies dealing with river water isotope studies on the southern Tibetan Plateau [26,27], precipitation isotope analyses in monsoon-dominated Tibetan Plateau regions [20], and leaf wax *n*-alkane δD measurements in Eastern China [28]. The observed lapse rate corresponds closely to the −0.13‰/100 m precipitation lapse rate previously reported for the monsoon Tibetan Plateau [20]. This phenomenon can be attributed to adiabatic cooling of rising water vapor, during which heavy isotopes preferentially precipitate due to their stronger molecular bonds [29].

Outside the ISM region, other Asian regions exhibit markedly different δ^18^O_TR__–_elevation patterns (Fig. S5). The EASM region shows a weak non-significant negative correlation (Fig. 4c, *r* = −0.10, *p* > 0.05, *n* = 102, Fig. 4c), while the arid westerlies region demonstrates a significant positive relationship (Fig. S5a, *r* = 0.30, *p* < 0.05, *n* = 52). High-latitude areas also display a weak non-significant negative relationship (Fig. S5b, *r* = −0.13, *p* > 0.05, *n* = 29). The unexpected positive δ^18^O_TR_–elevation relationship in arid westerlies region likely arises from the interaction of multiple moisture sources, enhanced contributions from recycled surface water, and sub-cloud evaporation processes [20,26,27]. These factors could overprint the original isotopic signal of source moisture and may obscure the expected negative δ^18^O-elevation gradient. Such positive isotope–elevation linkages are further supported by satellite-based observations of water vapor δD [30], river water δ^18^O analyses [26,27], and leaf wax *n*-alkane δD measurements [28] in Asia.

These region-specific δ^18^O_TR_–elevation linkages have significant implications for paleoelevation reconstructions. Specifically, isotope-based reconstructions of paleoelevation are most reliable in regions dominated by a single, consistent moisture source with substantial long-distance transport, such as the ISM system. Conversely, in areas influenced by multiple or variable moisture sources, isotope–elevation relationships may be biased and should therefore be interpreted with considerable caution.

### Regional differences for drivers of spatial δ^18^O_TR_ pattern

**Figure 5.**
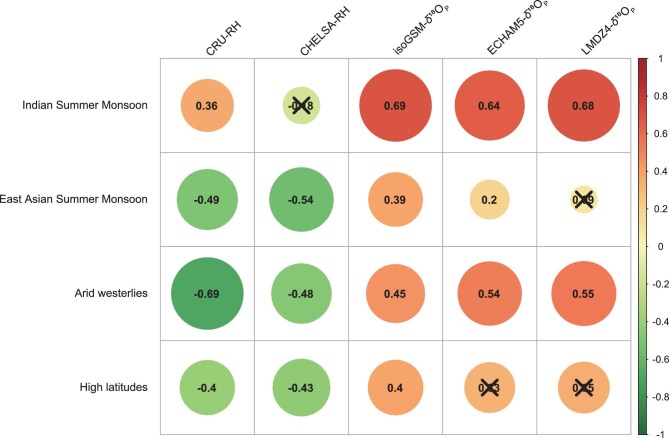
Pearson correlation coefficients between observed δ^18^O_TR_ and key drivers (RH from CRU and CHELSA, and simulated May–September δ^18^O_P_ from three isotope-enabled models) across four Asian subregions. Non-significant correlations (*p* > 0.05) are marked with ×; color intensity indicates correlation strength.

The drivers of observed δ^18^O_TR_ across Asia are region-specific, confirming our second hypothesis (Fig. 5). In the ISM region, δ^18^O_P_ emerges as the primary driver of δ^18^O_TR_ (*r* = 0.64–0.69, *p* < 0.05), followed by RH (CRU: *r* = 0.36, *p* < 0.05; CHELSA: *r* = −0.18, *p* > 0.05, Fig. 5). Commonality analysis further supports this pattern, revealing that δ^18^O_P_ consistently explains a larger proportion of δ^18^O_TR_ variance than RH (Fig. S6). Such dominant influence of δ^18^O_P_ on δ^18^O_TR_ has been supported by intra-annual isotope observations and process-based isotopic modelling on the southeastern Tibetan Plateau [10]. In the EASM region, RH demonstrates a stronger statistical relationship with δ^18^O_TR_ (CRU: *r* = −0.49, *p* < 0.05; CHELSA: *r* = −0.54, *P* > 0.05) compared to δ^18^O_P_ (ECHAM5: *r* = 0.2, *p* < 0.05; LMDZ4: *r* = 0.09, *p* > 0.05; isoGSM: *r* = 0.39, *p* < 0.05, Fig. 5), which is also supported by the results of commonality analysis (Fig. S7). Such finding is corroborated by δ^18^O_TR_ observational data and validated through process-based isotopic modeling approaches [31]. These may explain why δ^18^O_TR_ is often employed to reconstruct RH in the EASM region [32,33]. RH affects δ^18^O_TR_ mainly in two ways: (i) when RH is low, plants lose more water through their leaves, which makes the leaf water richer in ^18^O. This enriched water is used to make tree rings; and (ii) low RH also causes more evaporation from the soil, making the water taken up by roots richer in ^18^O. Both effects lead to higher δ^18^O_TR_ [6,32].

In the arid westerlies region, it seems challenging to disentangle the relative contributions of RH and δ^18^O_P_ to δ^18^O_TR_ due to their similar correlation coefficients (CRU-RH: *r* = −0.69, *p* < 0.05; CHELSA-RH: *r* = −0.48, *p* > 0.05; δ^18^O_P_: *r* = 0.45–0.55, *p* < 0.05, Fig. 5). Commonality analysis using CHELSA RH and δ^18^O_P_ from ECHAM5 and LMDZ4 revealed a stronger influence of δ^18^O_P_ on δ^18^O_TR_, whereas analyses using CRU RH and δ^18^O_P_ from IsoGSM, ECHAM5, and LMDZ4 indicated a greater contribution from RH (Fig. S8). Additionally, commonality analysis combining CHELSA RH and δ^18^O_P_ from IsoGSM also showed RH as the dominant driver (Fig. S8). Such results may be attributed to the high contribution of recycled moisture to local precipitation, which enhances the coupling between RH and δ^18^O_P_. Moreover, in the high latitude region, RH exhibits a slightly stronger overall influence on δ^18^O_TR_ (CRU: *r* = −0.40, *p* < 0.05; CHELSA: *r* = −0.43, *p* > 0.05) compared to δ^18^O_P_ (ECHAM5: *r* = 0.33, *p* > 0.05; LMDZ4: *r* = 0.35, *p* > 0.05; IsoGSM: *r* = 0.40, *p* < 0.05, Fig. 5). Commonality analysis only using CRU RH and δ^18^O_P_ from IsoGSM revealed a slightly greater contribution from δ^18^O_P_ to δ^18^O_TR_. In contrast, analyses using other combinations consistently indicated a higher contribution from RH than δ^18^O_P_ (Fig. S9). Such linkages may result from the influence of permafrost-affected source water, which can overwrite or obscure the climatic signals imprinted in δ^18^O_TR_ [34]. This may complicate the interpretation of δ^18^O_TR_ in high-latitude permafrost regions, where no single dominant climatic driver is evident (*r* < 0.6) [35,36].

### Applications

The development of the first Asian δ^18^O_TR_ isoscapes presented in this study opens up a wide range of research opportunities across paleoclimate and (paleo)environmental sciences. In paleoclimate research, these maps offer a valuable framework for reconstructing hydroclimatic variability, tracking monsoon dynamics. In ecological studies, δ^18^O_TR_ isoscapes can support investigations into wood provenance and migration. For hydrology, combining δ^18^O_TR_ with climate data enables improved tracking of water cycle changes, identification of moisture sources. Beyond these fields, potential applications include forensic timber tracing and archaeological artifact sourcing.

## SUMMARY AND OUTLOOK

In summary, to the best of our knowledge, this study represents the first attempt to generate δ^18^O_TR_ isoscapes at a continental scale for Asia, offering a foundational framework for exploring large-scale hydroclimatic processes and enhancing dendroprovenancing applications. The resulting isoscapes reveal a pronounced ‘sandwich’ pattern, characterized by depleted δ^18^O_TR_ values at high (>50°N) and low (<30°N) latitudes, and enriched values across the mid-latitudes (30°N–50°N). This spatial distribution closely aligns with May–September δ^18^O_P_ observed in GNIP and TNIP datasets. Our reconstructed δ^18^O_TR_ field, informed by δ^18^O_P_ outputs from global isotope-enabled models and RH, effectively reproduces this latitudinal gradient. We identify δ^18^O_P_ as the primary driver of δ^18^O_TR_ variability across the continent based on correlation and commonality analysis. Notably, the relative importance of these drivers varies regionally: δ^18^O_TR_ is primarily driven by δ^18^O_P_ in the ISM, by RH in the EASM and high-latitude regions, while in the arid westerlies the relative contributions of RH and δ^18^O_P_ are difficult to disentangle. While the δ^18^O_TR_–elevation relationship is negligible at the continental scale, substantial heterogeneity emerges at regional levels. A significant negative correlation is observed in the ISM domain, contrasting with weak or even positive correlations in the EASM, arid westerlies, and high latitude regions. These findings suggest that isotope-based paleoaltimetry may only be viable in regions dominated by consistent moisture sources and long-range atmospheric transport, such as the ISM. Collectively, our results underscore the utility of δ^18^O_TR_ isoscapes as proxies for reconstructing δ^18^O_P_ distributions, diagnosing shifts in atmospheric circulation (e.g. ISM variability), and informing dendroprovenancing analyses.

While this study offers valuable insights into the variability of δ^18^O_TR_ isoscapes, some limitations of this study should be acknowledged. First, we assumed that May–September δ^18^O_P_ could serve as a direct proxy for source water in modeling δ^18^O_TR_, without explicitly accounting for soil-related processes such as soil depth, composition evaporation, root uptake depth, and residence time of soil water. While this assumption may hold in humid regions, it is likely less valid in arid environments, where trees may rely on moisture accumulated prior to the growing season or derived from snowmelt [37]. Second, when reconstructing δ^18^O_P_ using δ^18^O_TR_ and RH (e.g., Fig. 1d), and generating modeled δ^18^O_TR_ isoscapes from RH and δ^18^O_P_ (e.g., Fig. 2), we treated several key parameters as constants (e.g., *f*_0_ = 0.42, ε* = 9‰, ε*_k_* = 29‰). However, these values are known to vary among species and even within individuals, which may introduce uncertainty into the modeled δ^18^O_TR_. Third, while our δ^18^O_TR_ isoscapes capture large-scale a “sandwich” pattern (Fig. 1b), caution is warranted when interpreting fine-scale features, particularly in heterogeneous landscapes. Fourth, our analysis is based on multi-year averaged δ^18^O_TR_ values, which capture the climatological patterns of δ^18^O_TR_ isoscapes but do not reflect interannual variability.

Consequently, building on the present findings, we suggest the following research directions need to be taken into account in future studies to strengthen the future applicability of isoscapes in the fields of ecology, hydrology, climate, geology, forensics: (i) global δ^18^O_TR_ isoscapes development—expand current efforts to a global scale by integrating more extensive datasets, improving spatial resolution and model fidelity; (ii) proxy-model integration and comparisons—combine δ^18^O_TR_ with δ^18^O_P_ isoscapes, model simulations [38–41], and other hydrological and ecological proxies (e.g. river water, tap water, leaf water) [4,42] to enhance multi-proxy reconstructions of environmental processes; (iii) temporal δ^18^O_TR_ isoscapes generation for the past 50–60 years—generate annually resolved δ^18^O_TR_ isoscapes to capture interannual variability over the past 50–60 years [2,5]; (iv) application of the δ^18^O_TR_ isoscapes for dendroprovenancing—utilize δ^18^O_TR_ isoscapes to enhance dendroprovenancing applications, building on the pioneering work in the USA [17] and Italy [18].

## MATERIALS AND METHODS

### Collection and preprocessing of δ^18^O_TR_ and δ^18^O_P_ data

We compiled 313 δ^18^O_TR_ records across Asia through systematic literature review [9], expanding the dataset through to April 2025. To standardize species differences, broadleaf δ^18^O_TR_ values were adjusted by +1.9‰ based on established fractionation differences between broadleaf and coniferous species [43,44]. δ^18^O_P_ data were obtained from 252 monitoring stations (233 from GNIP, 19 from TNIP [20]) collected in April 2023. May–September mean δ^18^O_P_ values were calculated for each site to align with the active vegetation period when trees record isotopic signals. We also reconstructed May–September δ^18^O_P_ using δ^18^O_TR_ and RH data through established transfer functions [7,8]. Due to the variability in both temporal resolution and record length across datasets, we chose not to report a single representative value in the abstract. Detailed information on the lengths of δ^18^O_TR_, GNIP, and TNIP records, as well as the equations used, is provided in the Supplementary Material (Figs S10–S14).

### δ^18^O_TR_ isoscape generation

Building on studies that apply machine learning to isoscape generation [2,5], we also employed XGBoost and Random Forest to produce 0.5°×0.5° δ^18^O_TR_ isoscapes using our compiled δ^18^O_TR_ data, along with a suite of geographic, climatic, and categorical variables. These include longitude, latitude, elevation, climate data and climate classifications. Isoscapes were extended to non-forested regions for comparison with δ^18^O_P_ distributions. Details are provided in the supporting materials (Figs S15–S18, Tables S2–S4).

### Modelling δ^18^O_TR_ isoscapes

For comparison, we modeled δ^18^O_TR_ spatial patterns using δ^18^O_P_ simulations from three validated isotope-enabled general circulation models (isoGSM [41], ECHAM5 [40] and LMDZ4 [39]) and RH data from CRU TS 4.07 [21] and CHELSA [45] datasets. RH was calculated using following equations [46]:


(1) \begin{eqnarray*}
{e}_{sat} = 6.108 \cdot {\mathrm{exp}}\left( {\frac{{17.27 \cdot {T}_{{mean}}}}{{{T}_{{mean}} + 237.3}}} \right),
\end{eqnarray*}

(2) \begin{eqnarray*}
{\rm RH} = 100 \cdot \frac{{\rm VAP}}{{{e}_{sat}}},
\end{eqnarray*}

where *T_mean_* and VAP represent monthly mean temperature and vapor pressure, respectively.

In calculating δ^18^O_TR_ from the simulated δ^18^O_P_ and RH in Figure 2, we interpolated the spatial resolution of the simulated δ^18^O_P_ to match the resolution of RH. The equation used to model δ^18^O_TR_ is provided in the Supplementary data, with parameter settings identical to those used for reconstructing δ^18^O_P_ in Fig. 1d.

### δ^18^O_TR_–elevation relationships

Elevation–δ^18^O_TR_ relationships were examined using elevation data from original publications or extracted from the ETOPO (Earth Topography) Global Relief model. Analysis was conducted at the continental scale and within four subregions: Indian Summer Monsoon, East Asian Monsoon, arid westerlies and high latitudes. Details about each of the defined subregions are provided in Supplementary data. Moreover, we examined the relationships between δ^18^O_TR_ and geographic position (longitude and latitude). Our results show that spatial position—particularly latitude—exerts a measurable influence on δ^18^O_TR_ across Asia (Fig. S19), consistent with the “sandwich” pattern observed in the isoscape. As the illustrative figure provides limited additional information and largely overlaps with the main figures (e.g., Fig. 1b), it is presented in the supplementary materials rather than in the main text to maintain brevity.

### Driving factors on δ^18^O_TR_ isoscapes in Asia and subregions

To elucidate the influence of δ^18^O_P_ and RH on δ^18^O_TR_ isoscapes, we extracted mean May–September δ^18^O_P_ and RH values for 313 tree-ring δ^18^O_TR_ sampling sites. Scatter plots were constructed to explore bivariate relationships between δ^18^O_TR_ and both δ^18^O_P_ and RH. Additionally, to quantitatively assess the individual and shared contributions of these environmental variables to spatial variation in δ^18^O_TR_, we employed commonality analysis [47]. Using a similar scatter plot, we investigated the relationships between δ^18^O_TR_ and both δ^18^O_P_ and RH within each subregion.

All analyses were performed using R version 4.4.3 [48].

## Supplementary Material

nwaf481_Supplemental_File

## Data Availability

The tree-ring oxygen isoscapes generated in this study (Fig. 1b) are available on Zenodo: https://zenodo.org/records/17503284. Model outputs from LMDZ4, ECHAM5 and isoGSM were obtained from the following repositories: (i) SWING2 Project Database: https://data.giss.nasa.gov/swing2/; (ii) Zenodo Repository: https://zenodo.org/records/1249604; and (iii) University of Tokyo Database: https://isotope.iis.u-tokyo.ac.jp/∼kei/tmp/isogsm2/flxmon.iso-n.nc. The CRU and CHELSA climate datasets are available at: (i) CRU: https://crudata.uea.ac.uk/cru/data/hrg/cru_ts_4.07/; (ii) CHELSA: https://chelsa-climate.org/downloads/. Digital Elevation Model (DEM) data (ETOPO_2022_v1_60s_N90W180) are available from NOAA: https://www.ncei.noaa.gov/products/etopo-global-relief-model. The GNIP dataset is accessible at https://www.iaea.org/services/networks/gnip. The TNIP dataset is provided by National Tibetan Plateau Data Center http://data.tpdc.ac.cn/en.
