# Supplementary material for: Asian continental isoscapes of tree-ring δ18O: implications for paleoclimate and paleoenvironment
Source: Natl Sci Rev. 2025 Nov 7;12(12):nwaf481. doi: 10.1093/nsr/nwaf481 (PMC12713636; doi:10.1093/nsr/nwaf481)
Supplement: nwaf481_Supplemental_File [file nwaf481_supplemental_file.docx]

Supplementary information

**Asian Continental Isoscapes of Tree-Ring δ¹⁸O: Implications for Paleoclimate and Paleoenvironment**

Ru Huang^1,3^, Chenxi Xu^1,2^*, Wenling An^1^, Qingyu Zhao^1^, Yaru Zhao^1^, Yucheng Liu^1,2^, Jussi Grießinger^3^, Wolfgang Jens-Henrik Meier^3^, Zhengtang Guo^1,2^

^1^State Key Laboratory of Lithospheric and Environmental Coevolution, Institute of Geology and Geophysics, Chinese Academy of Sciences, Beijing 100029, China

^2^College of Earth and Planetary Sciences, University of Chinese Academy of Sciences, Beijing 100049, China

^3^Department of Environment and Biodiversity, University of Salzburg, Salzburg, 5020, Austria

Corresponding author: Chenxi Xu, [cxxu@mail.iggcas.ac.cn](mailto:cxxu@mail.iggcas.ac.cn)

**Supplementary Text**

**Reconstructed May**–**September mean δ¹⁸O_P_**

We reconstructed the May–September mean δ¹⁸O_P_ based on δ¹⁸O_TR_ and May–September RH, using a transfer function derived from tree-ring isotope models:

$\delta^{18}O_{TR}=\delta^{18}O_{P}+\varepsilon_{0}+\alpha(1-f_{0})(1-RH)(\varepsilon^{*}+\varepsilon_{k})$ equation S1

$\delta^{18}O_{P}= \delta^{18}O_{TR}-\varepsilon_{0}-\alpha(1-f_{0})(1-RH)(\varepsilon^{*}+\varepsilon_{k})$ equation S2

The δ^18^O_P_ represents the δ^18^O value of precipitation, while *ε_0_* denotes the net biological isotopic fractionation factor between xylem water and exchanged oxygen in carbohydrates (set at 27%). The parameter ƒ_0_ indicates the fraction of isotopic exchange between xylem water and carbohydrate oxygen that remains temperature-independent (0.42). The equilibrium and kinetic isotopic fractionation factors are represented by *ε** and *ε_k_*, with respective values of 9‰ and 29‰. RH corresponds to relative humidity, and α represents the fraction of enriched water (assigned a value of 1). These parameter values are based on previously published studies [1-4].

**Isoscape Generation Using Machine Learning Approaches**

We employed two machine learning algorithms—XGBoost and Random Forest—to generate 0.5° × 0.5° δ¹⁸O_TR_ isoscapes using our compiled tree-ring oxygen isotope data alongside a comprehensive set of environmental predictors. An overview of the procedures is provided in Fig. S15. The modeling workflow comprised the following steps:

1. **Data Preparation** The input dataset included gridded climate, environmental, and categorical variables such as longitude, latitude, elevation, monthly and annual climate covariates (from CHELSA, CRU, and NCEP), and Köppen climate classifications (one-hot encoded). Coordinates from 313 tree-ring sampling sites and 21,500 CRU grid points were used to extract variables from the CHELSA, CRU, and NCEP datasets, with averages computed for the May–September period. Data were split into training samples (n = 313, based on tree-ring δ¹⁸O data) and prediction grid points (n = 21,500, based on CRU gridded coverage across Asia). Details about the data are provided in Table S2 and Fig. S16.
2. **Variable Set Construction** Multiple predictor sets were constructed to evaluate the contribution of different variable combinations, including geographic coordinates, elevation, individual and aggregated climate variables, and categorical climate zones. Variable indices and names were stored as lists to enable flexible subset selection.
3. **Hyperparameter Tuning and Model Training (XGBoost)** For each predictor set, XGBoost regression models were trained using 5-fold cross-validation and randomized hyperparameter search (30 candidate configurations, adjustable based on computational resources). The best-performing hyperparameters were selected and used to train the final model on the full training dataset.
4. **Model Evaluation (XGBoost)** Model performance was assessed using standard accuracy metrics: mean squared error (MSE), root mean squared error (RMSE), mean absolute error (MAE), and coefficient of determination (R²). Evaluation results were exported as CSV files for each predictor configuration.
5. **Variable Importance (XGBoost)** Feature importance was quantified using XGBoost’s internal ranking metrics and exported for further analysis.
6. **Spatial Prediction and Output (XGBoost)** The optimized XGBoost model was applied to all prediction grid points. Predicted isotope values were mapped to geographic raster grids and exported in NetCDF format for visualization and spatial analysis.
7. **Hyperparameter Tuning and Model Training (Random Forest)** In parallel, Random Forest regression models were trained using 5-fold cross-validation and randomized grid search over 1,500 candidate mtry values, with 500 trees per model. Optimal hyperparameters were selected and used to train the final Random Forest models.
8. **Model Evaluation (Random Forest)** Random Forest performance was evaluated using the same metrics (MSE, RMSE, MAE, R²), and summary statistics were exported for each configuration.
9. **Variable Importance (Random Forest)** Feature importance was assessed using the mean decrease in node impurity and exported for comparative analysis.
10. **Spatial Prediction and Output (Random Forest)** The optimized Random Forest model was used to generate gridded predictions, which were also saved in NetCDF format.

All above procedures were implemented in R version 4.4.3 [5], employing the packages *xgboost* [6] and *randomForest* [7]. Moreover, all model outputs—including prediction rasters, accuracy metrics, and variable importance rankings—were systematically exported to ensure reproducibility and facilitate further evaluation.

**Methodological Distinctions from Previous Studies about the machine learning approaches**

Parameter selection was guided by two relevant studies [8, 9]. However, our approach diverges in several key aspects:

1. **Exclusion of Teleconnection Indices** Unlike the referenced studies, which focus on monthly-scale climate variability, our objective was to construct climatological maps. Large-scale circulation indices exert limited influence on long-term climatological indicators and were therefore excluded from our analysis.
2. **Use of High-Resolution Climate Data** While previous studies relied on ERA5 data at ~31 km resolution, we utilized CHELSA data at ~1 km resolution to improve spatial accuracy.
3. **Handling of Missing Data** Given the sensitivity of machine learning models to missing values, we excluded any variable containing missing data. With a sufficiently large set of predictors (n = 102), occasional gaps in a few variables did not significantly affect model performance. The final models consistently achieved excellent fit, with R² values exceeding 0.9 (Tables S3–S4).
4. **Variable Selection Relative to Nelson et al. (2021)** We did not include the following variables used in Nelson et al.: NCEP-downward longwave flux at surface, NCEP-downward shortwave flux at surface, NCEP-low cloud top pressure, and NCEP-middle cloud bottom pressure. The first two variables were unavailable, and the latter two contained missing values across the 313 tree-ring sampling sites.

**Evaluation of Isoscapes Across Approaches and Parameter Combinations**

We visualized all isoscapes generated from two machine learning approaches (XGBoost and Random Forest) under various parameter combinations (Fig. S17–18). Despite methodological differences, all outputs consistently exhibit a "sandwich" pattern: lower δ¹⁸O_TR_ values at high (>50°N) and low latitudes (<30°N), and elevated values across mid-latitudes (30–50°N). Based on model performance (Tables S3–S4), characterized by low RMSE and high R² values, we selected the XGBoost isoscape derived from the “Longitude–Latitude–Elevation–CHELSA–Climate Zone” parameter set for presentation in the main text. All other isoscapes and comparative results are provided in the supporting materials.**Resolution mismatch and sensitivity analysis**

The resolution mismatch between tree-ring sampling sites (hundreds of metres) and gridded climate datasets (tens to hundreds of kilometres) may introduce uncertainty, particularly in topographically complex regions. To assess this effect, we compared four approaches: (1) thin-plate spline regression with latitude and longitude; (2) XGBoost with geographic and climatic zone predictors; (3) XGBoost with CRU variables (0.5° × 0.5°); and (4) XGBoost with CHELSA variables (30-arcsecond). All methods reproduced the large-scale “sandwich” pattern of δ¹⁸O_TR_, but machine learning approaches provided clearer spatial structures than interpolation (Fig. S20). Incorporating high-resolution climate data, especially CHELSA, improved the representation of local variability. These results indicate that continental-scale gradients are robust, but fine-scale patterns should be interpreted with caution in heterogeneous landscapes.

**Definition of four subregions**

High latitude region was defined as sites located at or above 60°N. The Indian Summer Monsoon (ISM) and East Asian Summer Monsoon (EASM) regions were delineated according to definitions provided in the latest IPCC report (<https://github.com/IPCC-WG1/Atlas/tree/main/reference-regions>). The arid westerlies region was characterized by an aridity index below 0.5, based on Version 3 of the "Global Aridity Index and Potential Evapotranspiration (ET0) Database"[10]. Our resulting dataset comprises 47 sites in the arid westerlies region, 29 sites in high latitudes, 88 sites in the Indian Summer Monsoon region, and 99 sites in the East Asian Summer Monsoon region (Supplementary Fig. 4).

**Supporting figures (20 figures)**


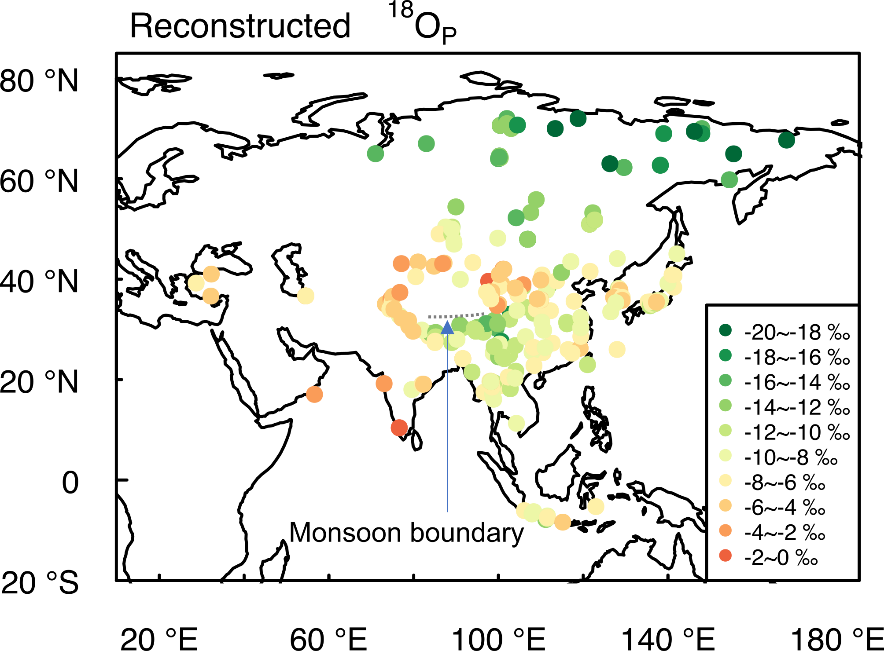


**Fig. S1** Reconstructed May–September δ¹⁸O_P_ across Asia (313 sites). We used δ¹⁸O_TR_ and May–September relative humidity from CHELSA 2.1 (1981–2010) [11] and transfer functions from isotope models[1, 2]. The black dotted lines represent the boundary of the Indian summer monsoon.


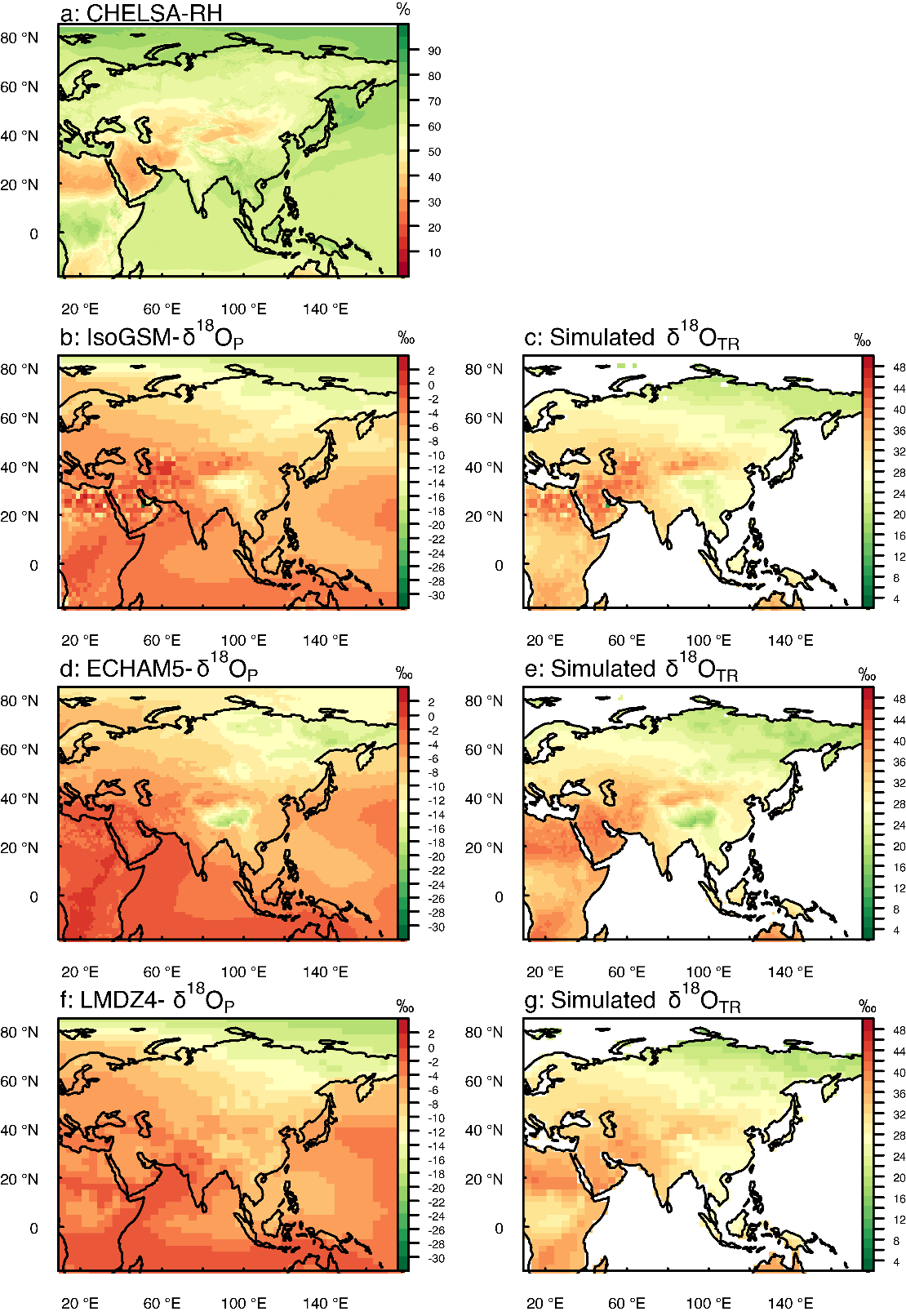


**Fig. S2** Spatial distribution of relative humidity and model-simulated δ¹⁸O_P_ and δ¹⁸O_TR_ across Asia. **a** Mean May–September relative humidity from the CHELSA dataset. **b**, **d**, **f** Simulated δ¹⁸O_P_ for the same period from three global isotope-enabled climate models: IsoGSM (**b**), ECHAM5 (**d**), and LMDZ4 (**f**). **c**, **e**, **g** Corresponding simulations of δ¹⁸O_TR_ from each respective model. The figure illustrates consistent spatial gradients and inter-model variations in both precipitation isotopes and tree-ring signals.


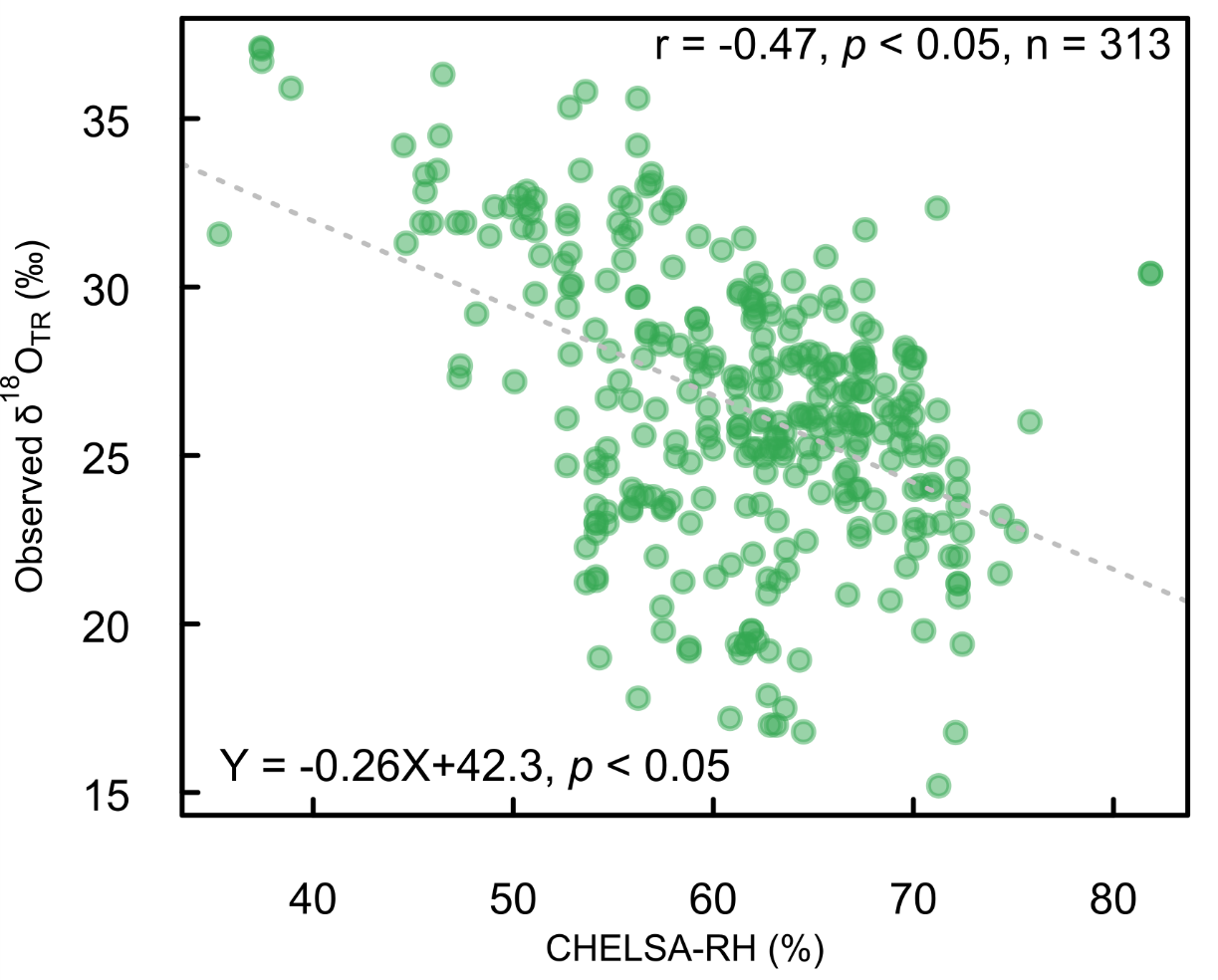


**Fig. S3** Relationship between δ¹⁸O_TR_ and CHELSA relative humidity. Scatter plot showing observed δ¹⁸O_TR_ versus CHELSA relative humidity for 313 δ¹⁸O_TR_ sites. Pearson correlation coefficient is displayed in the upper right corner.


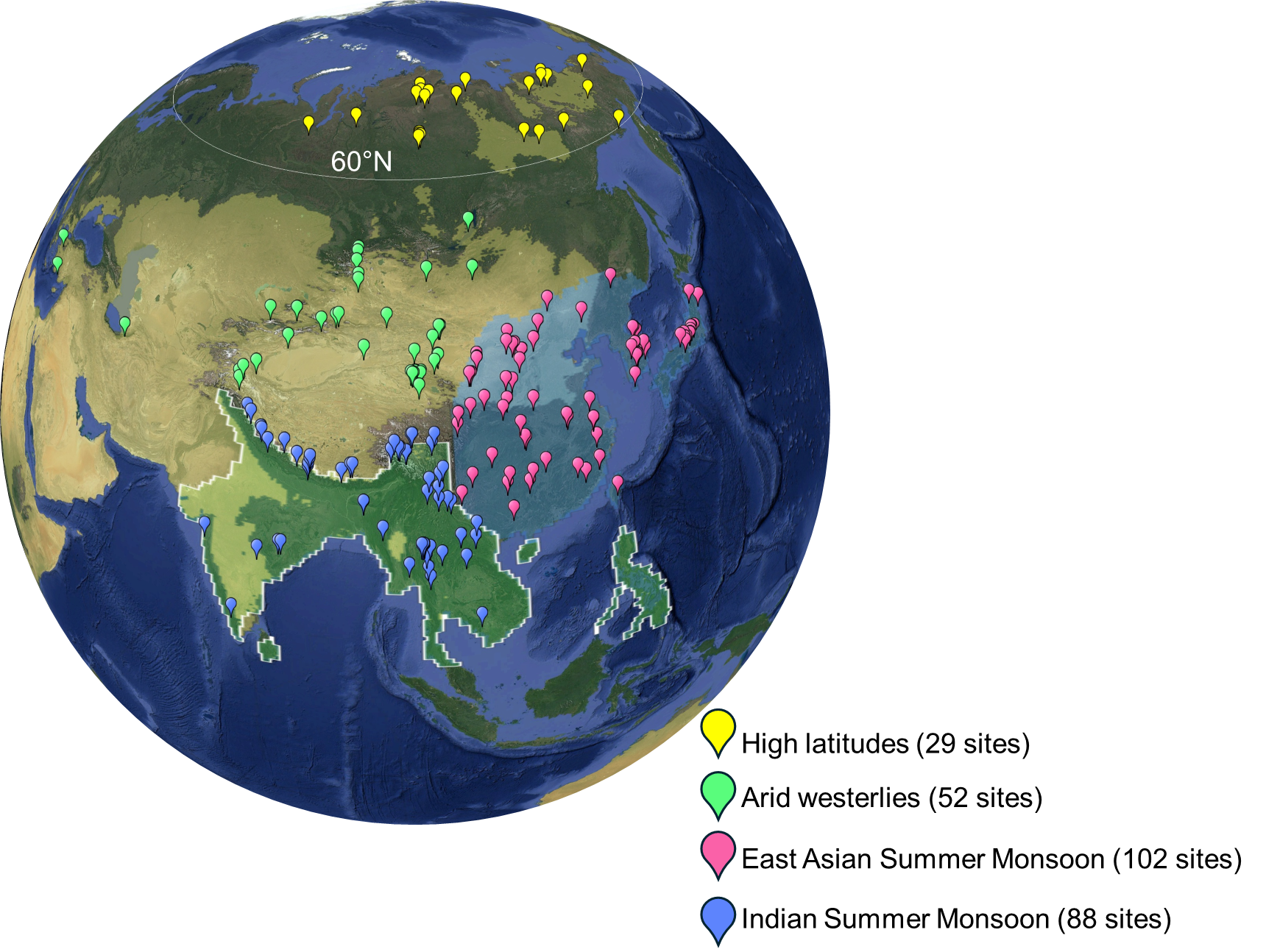


**Fig. S4** Geographic categorization of δ¹⁸O_TR_ sites into climate subregions. a Arid westerlies, b High latitudes, c Indian Summer Monsoon, and d East Asian Summer Monsoon. High latitude sites are defined as locations at or above 60°N. The Indian Summer Monsoon and East Asian Summer Monsoon regions are delineated following IPCC AR6 reference region definitions (<https://github.com/IPCC-WG1/Atlas/tree/main/reference-regions>). Arid westerlies are defined as areas outside the aforementioned monsoon and high-latitude regions, with an aridity index below 0.5, based on the Global Aridity Index and Potential Evapotranspiration Database v3[10]. Sites outside these four subregions were excluded from analysis. Base map: Google Earth, © Google, 2021.


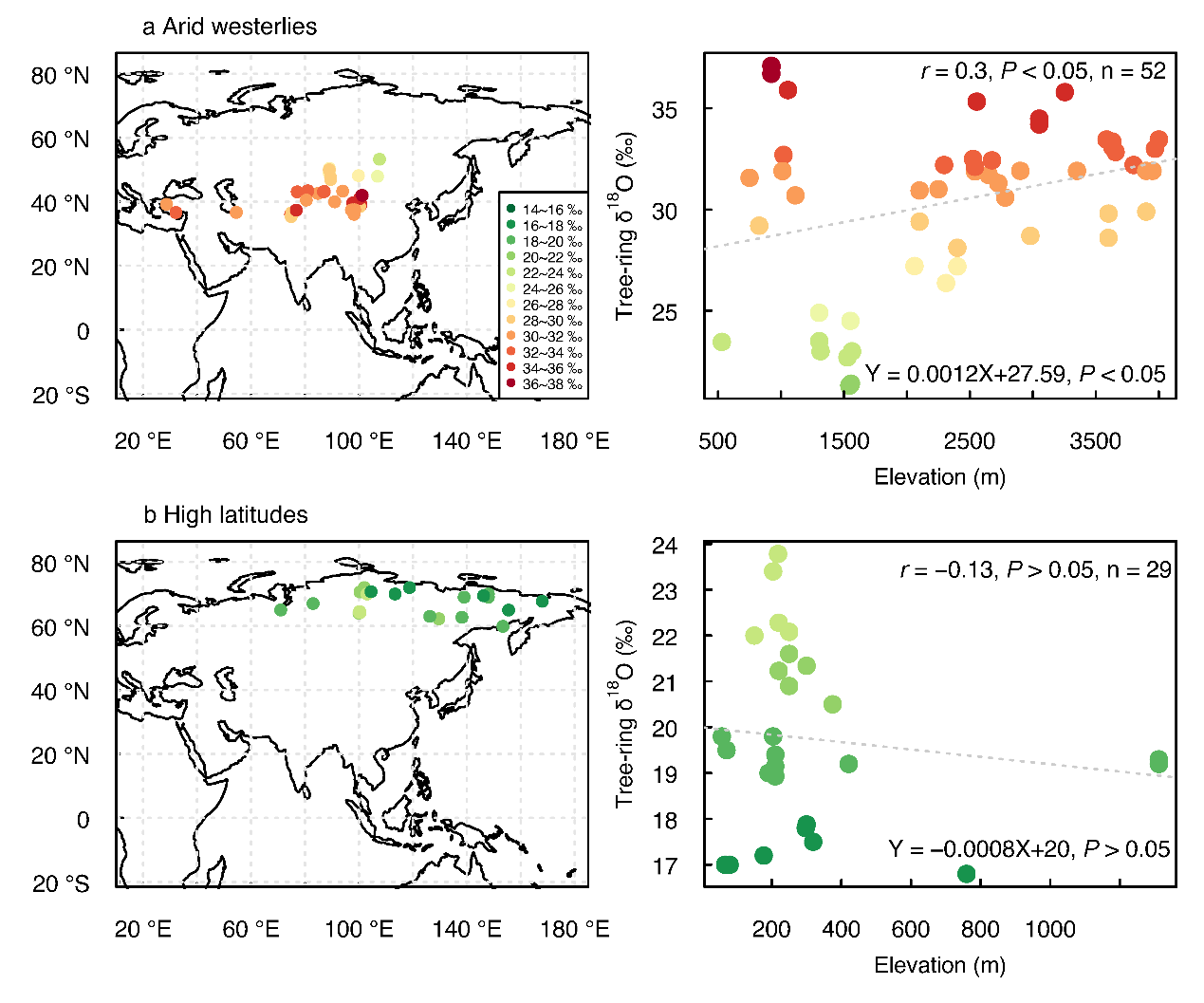


**Fig. S5** Regional δ¹⁸O_TR_-elevation relationships across different regions of Asia. **a** Arid westerlies regions. **b** High latitude regions. Left panels show spatial distributions of sampling sites; right panels show corresponding scatter plots of δ¹⁸O_TR_ versus elevation. Pearson correlation coefficients are displayed in the upper right corner of each scatter plot.


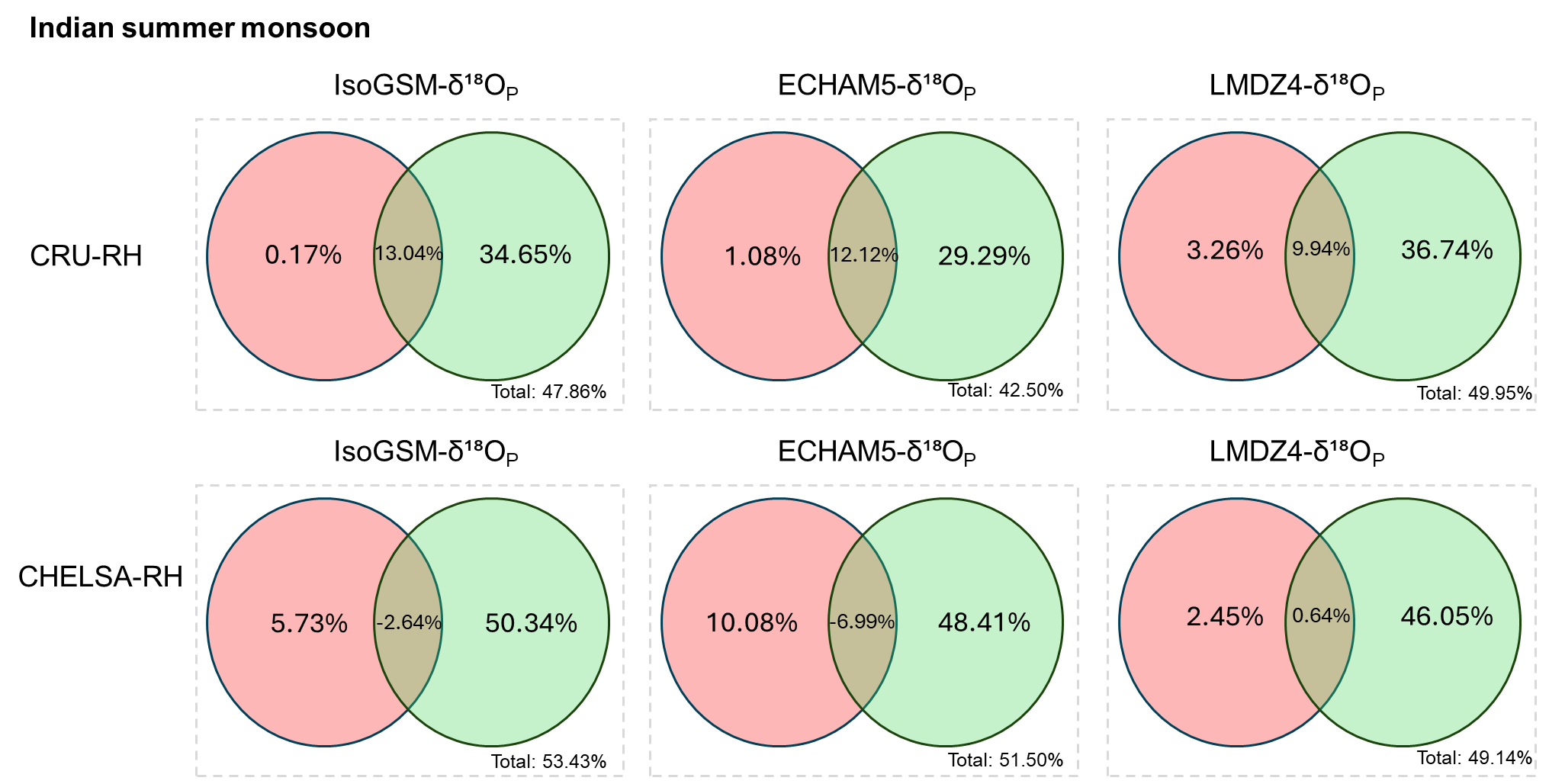


**Fig. S6** Venn diagrams illustrating the proportional overlap between three categories across six scenarios for the Indian summer monsoon region. Each diagram displays the percentage of elements unique to the red (relative humidity) and green areas (δ¹⁸O_P_), as well as their shared intersection (brown).


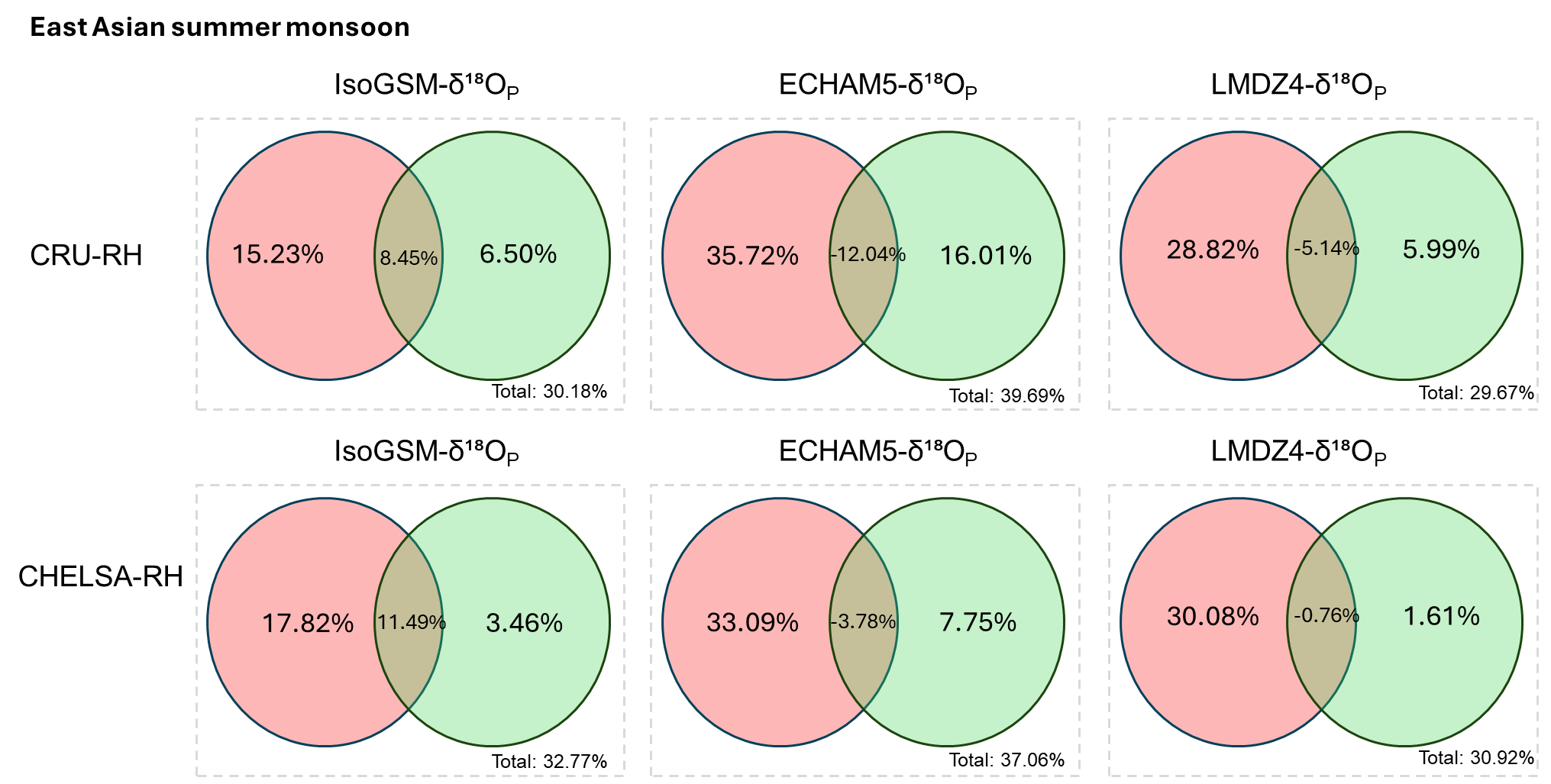


**Fig. S7** Same as the above figure but for the East Asian summer monsoon region.


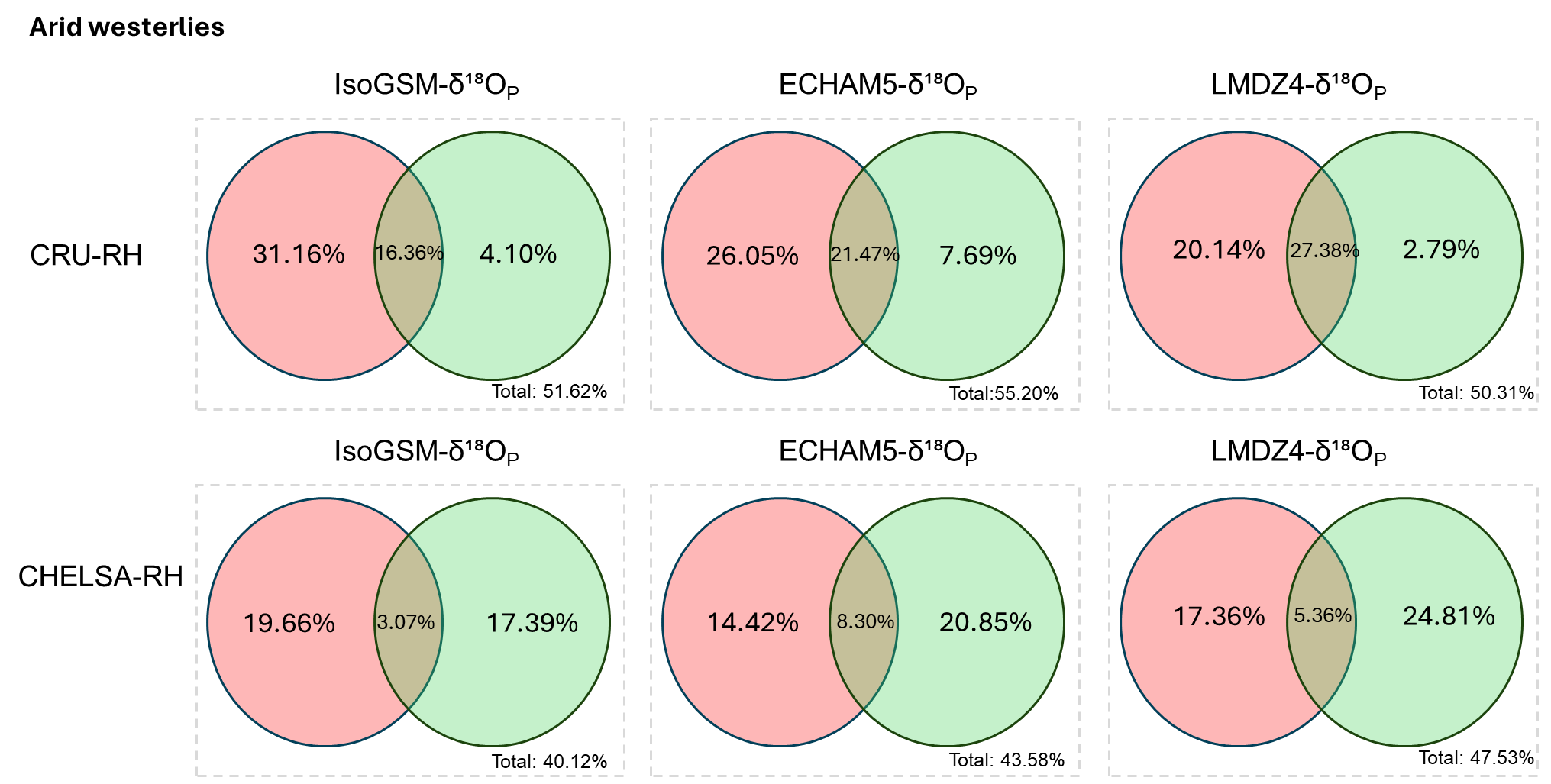


**Fig. S8** Same as the above figure but for the arid westerlies region.


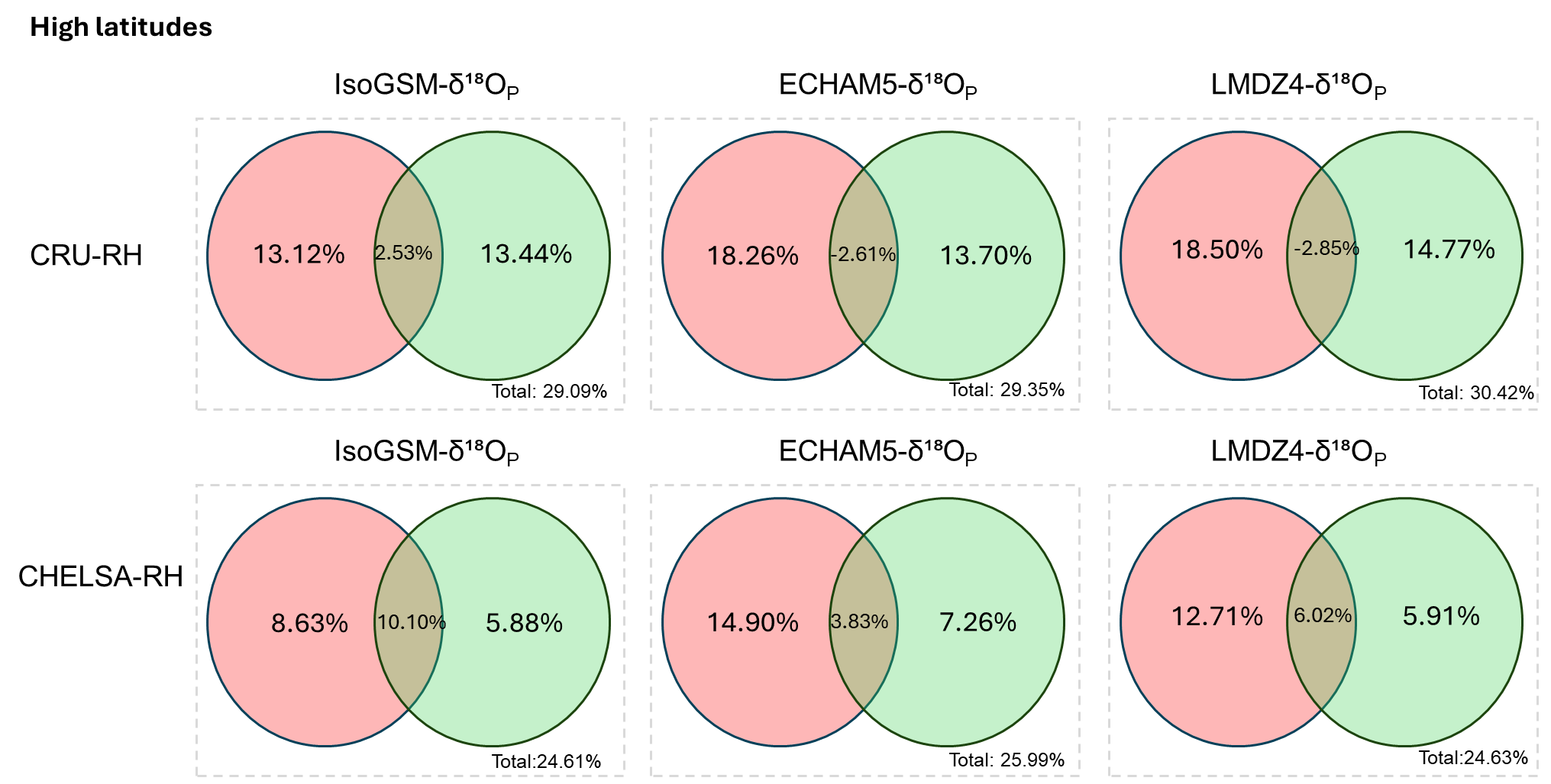


**Fig. S9** Same as the above figure but for the high latitudes region.


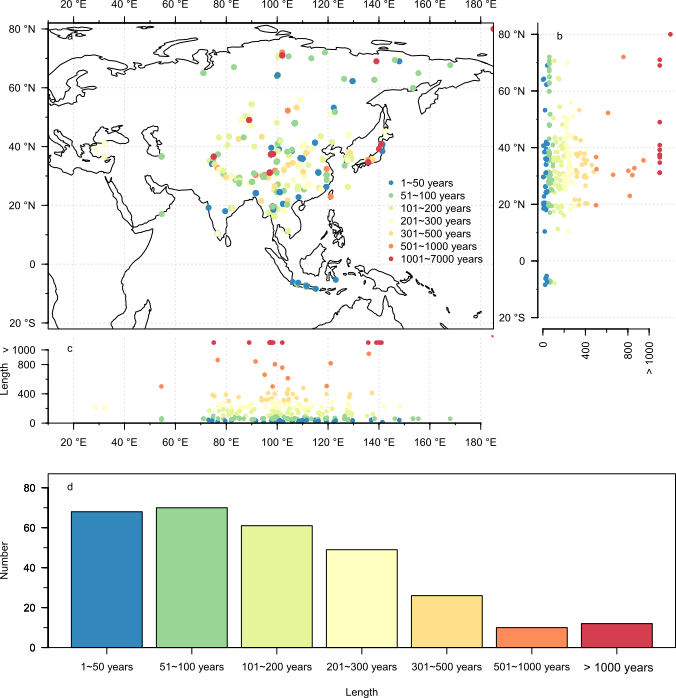


**Fig. S10**(a) Spatial distribution of the lengths of δ^18^O_TR_ chronologies in Asia. (b) Latitudinal pattern. (c) Longitudinal pattern. (d) Frequencies of different age classes.


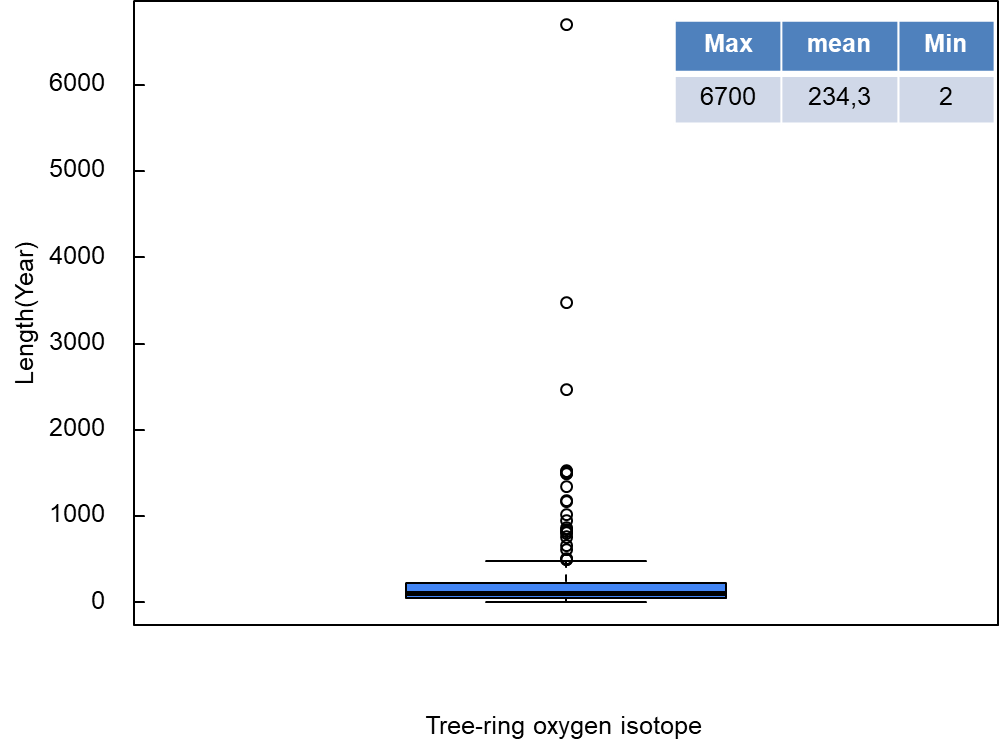


**Fig. S11** Boxplot for the length of 313 tree-ring oxygen isotope used in this work**.**


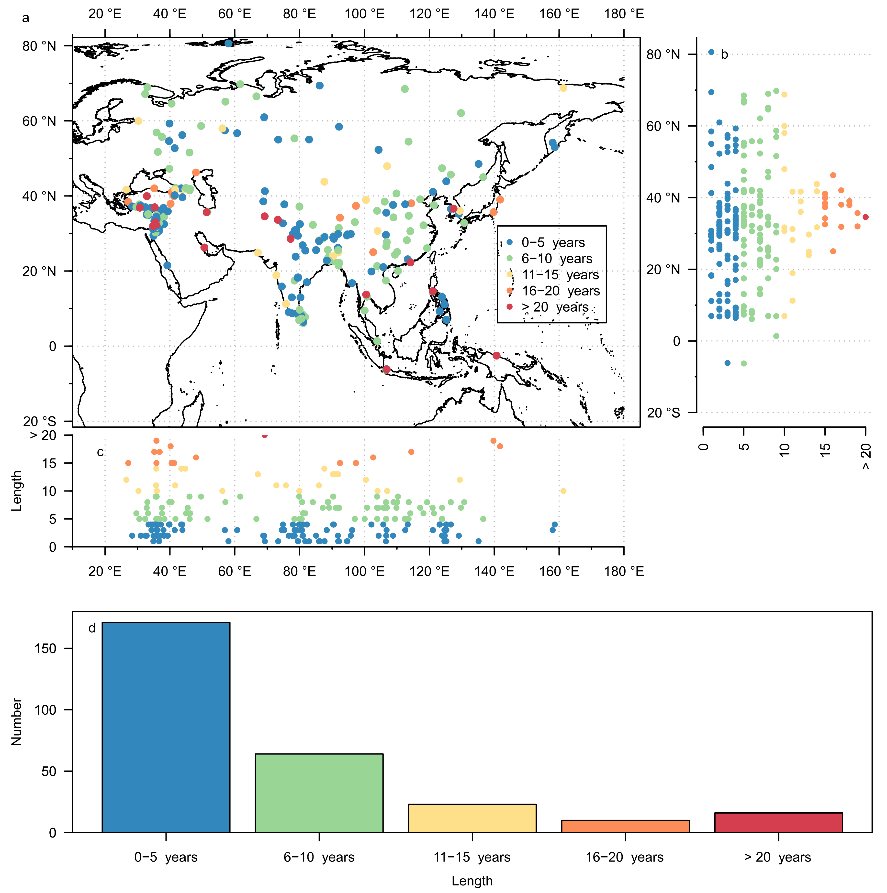


**Fig. S12** (a) Spatial distribution of the lengths of 252 GNIP/TNIP observations in Asia. (b) Latitudinal pattern. (c) Longitudinal pattern. (d) Frequencies of different length classes. In total, 289 GNIP/TNIP stations were compiled; 37 stations were excluded due to missing data during the May–September period.


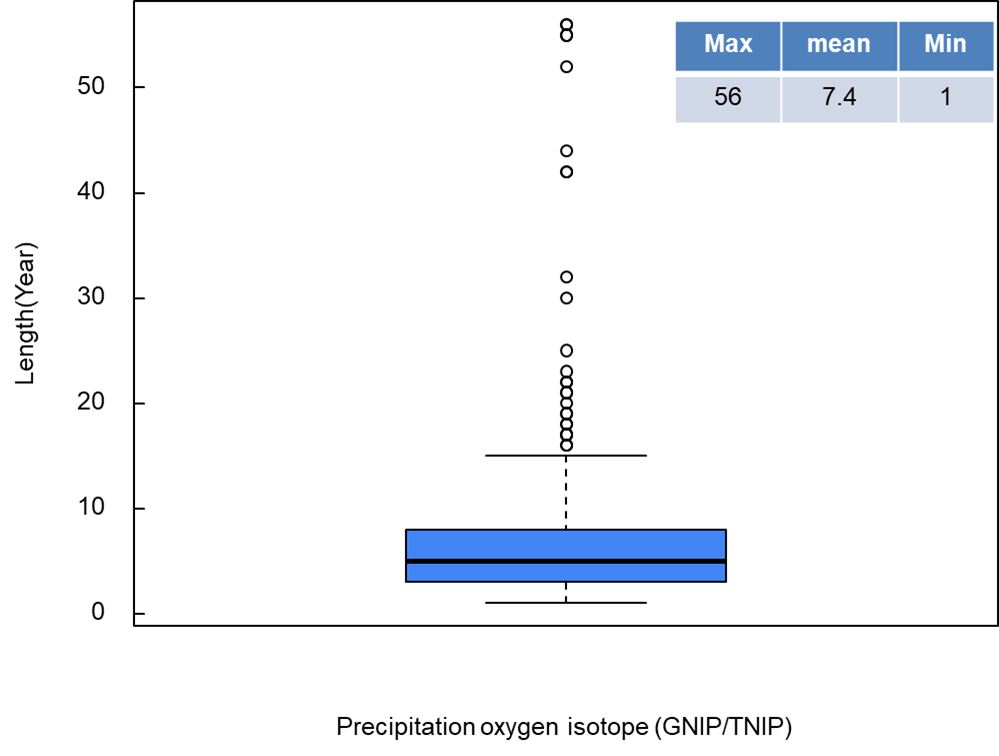


**Fig. S13** Boxplot for the length of precipitation oxygen isotope used in this work (GNIP and TNIP).


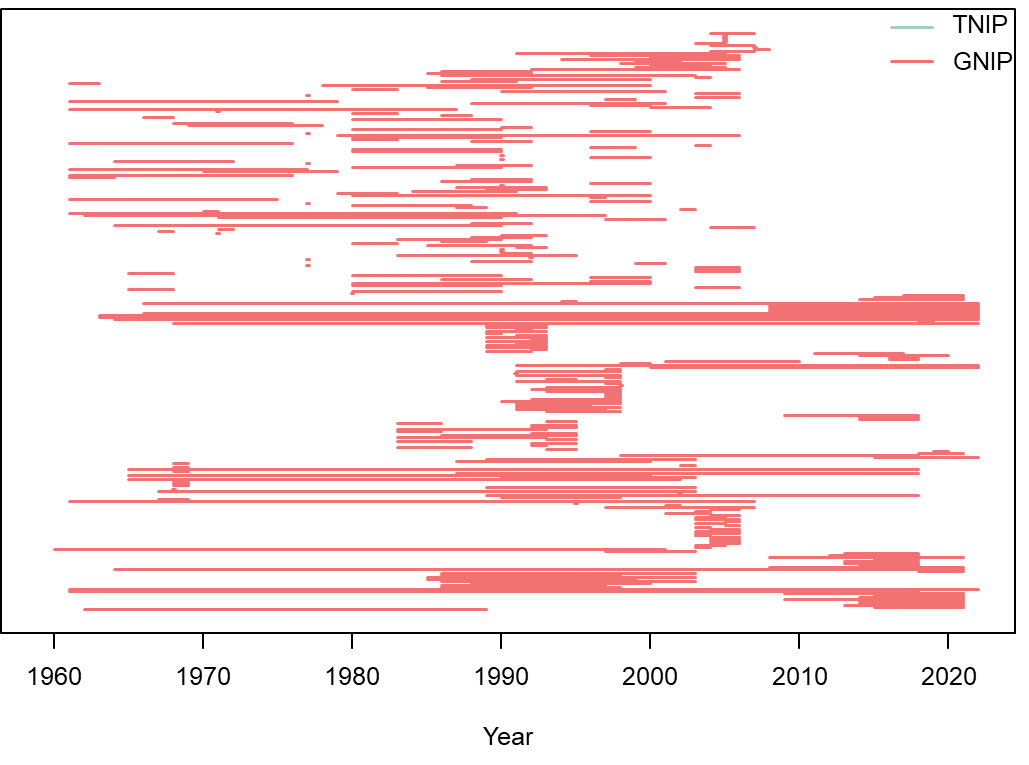


**Fig. S14** Temporal coverage of precipitation oxygen isotope datasets from GNIP (red) and TNIP (green) stations between 1960 and 2023. Each horizontal line represents the duration of data collection at individual stations, illustrating the variability in record lengths and the periods of overlap between GNIP and TNIP datasets.


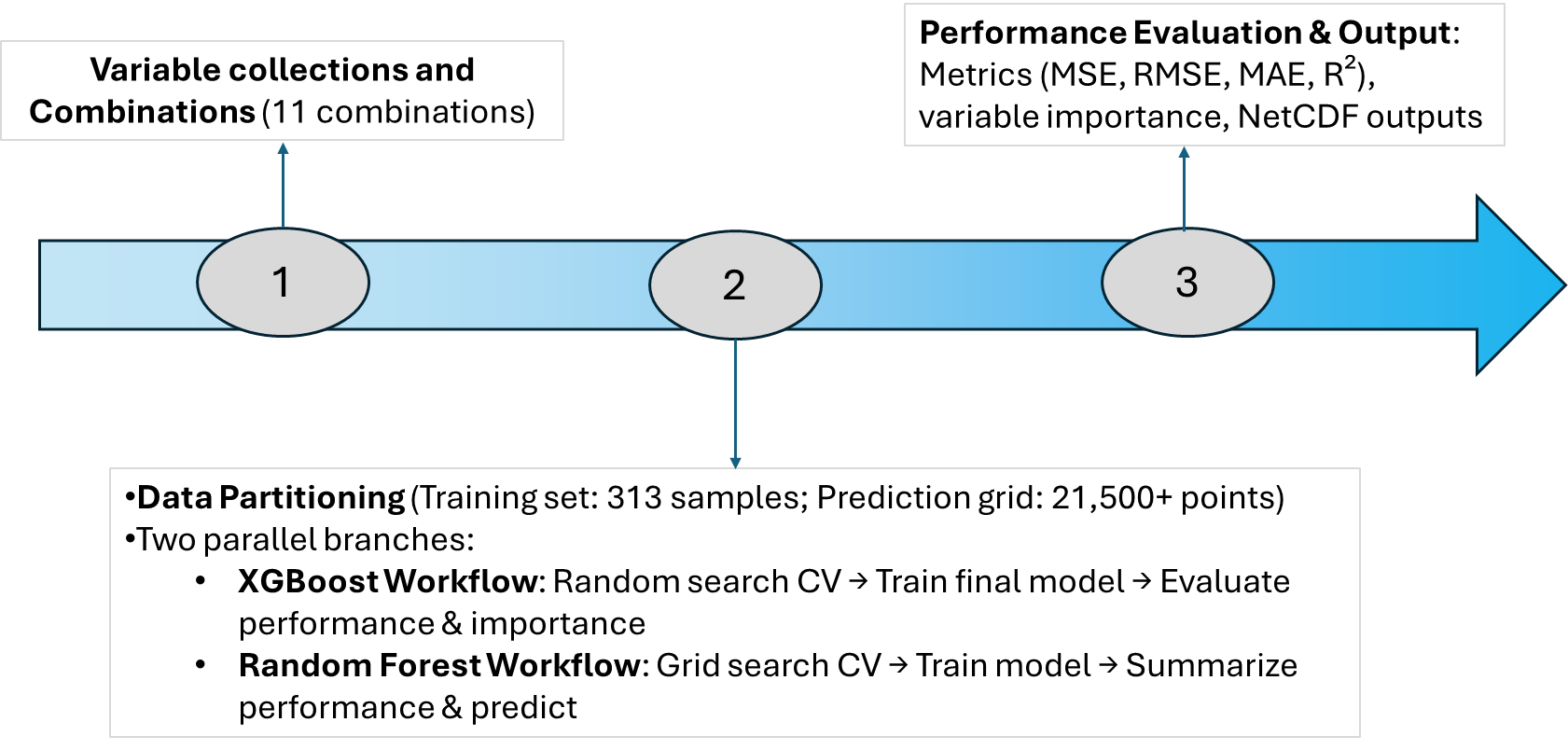


**Fig. S15** Steps to produce the isoscape using the machine approaches.


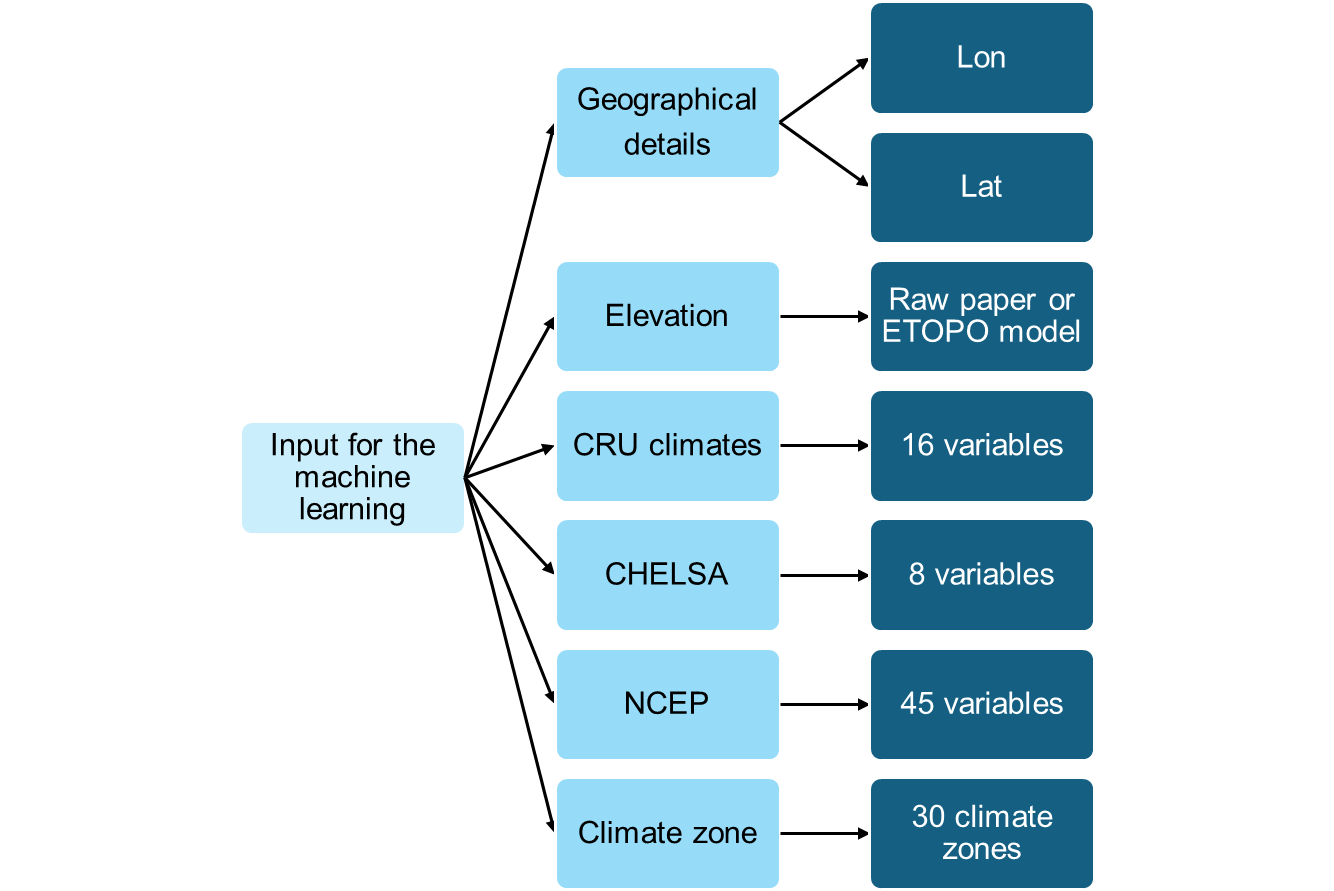


**Fig. S16** Variables used to produce the isoscape using the machine learning approaches.


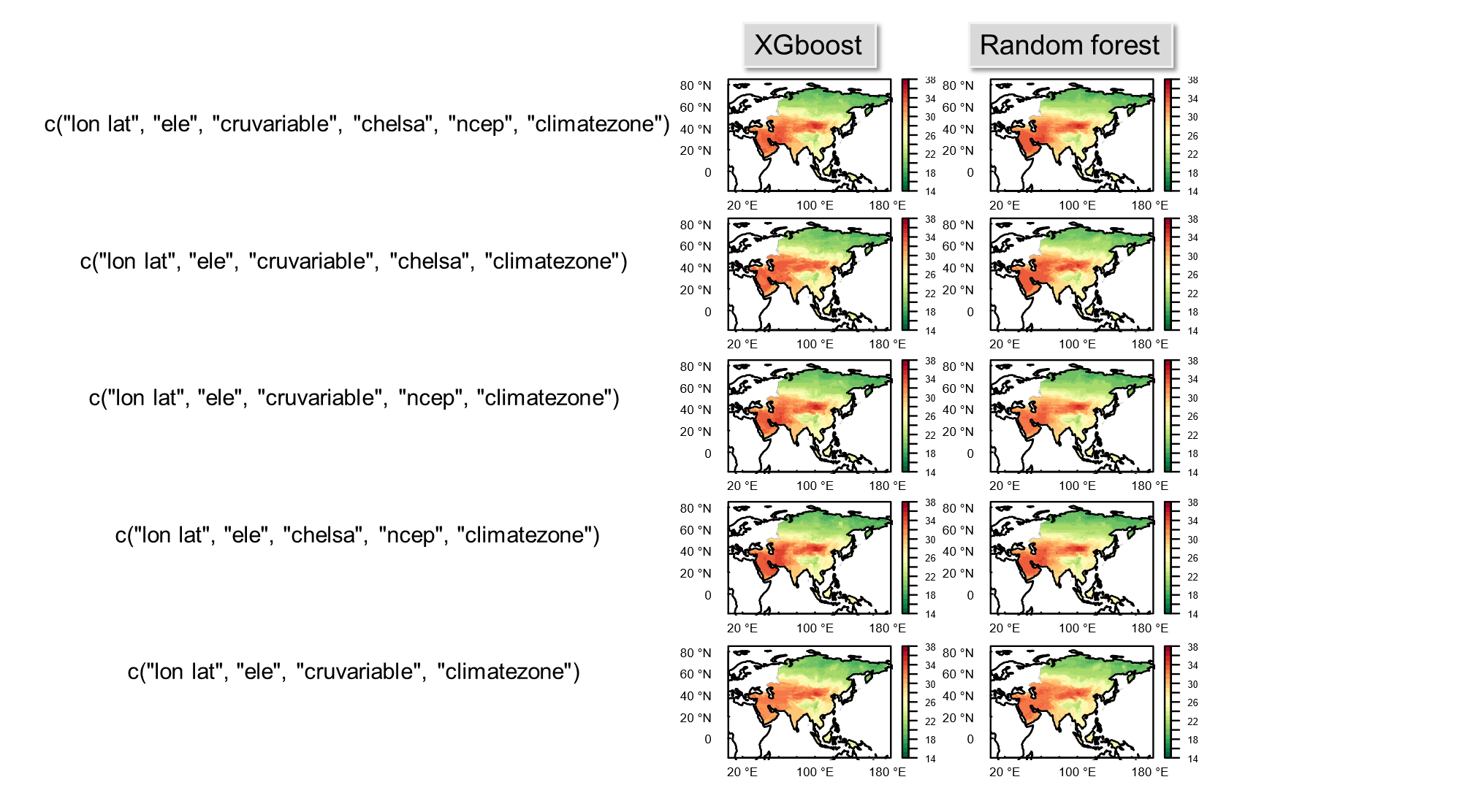


**Fig. S17** δ¹⁸O_TR_ isoscape based on XGBoost (left) and Random forest (right). Different rows stand for different combinations of parameters input (shown in the left of the figure)


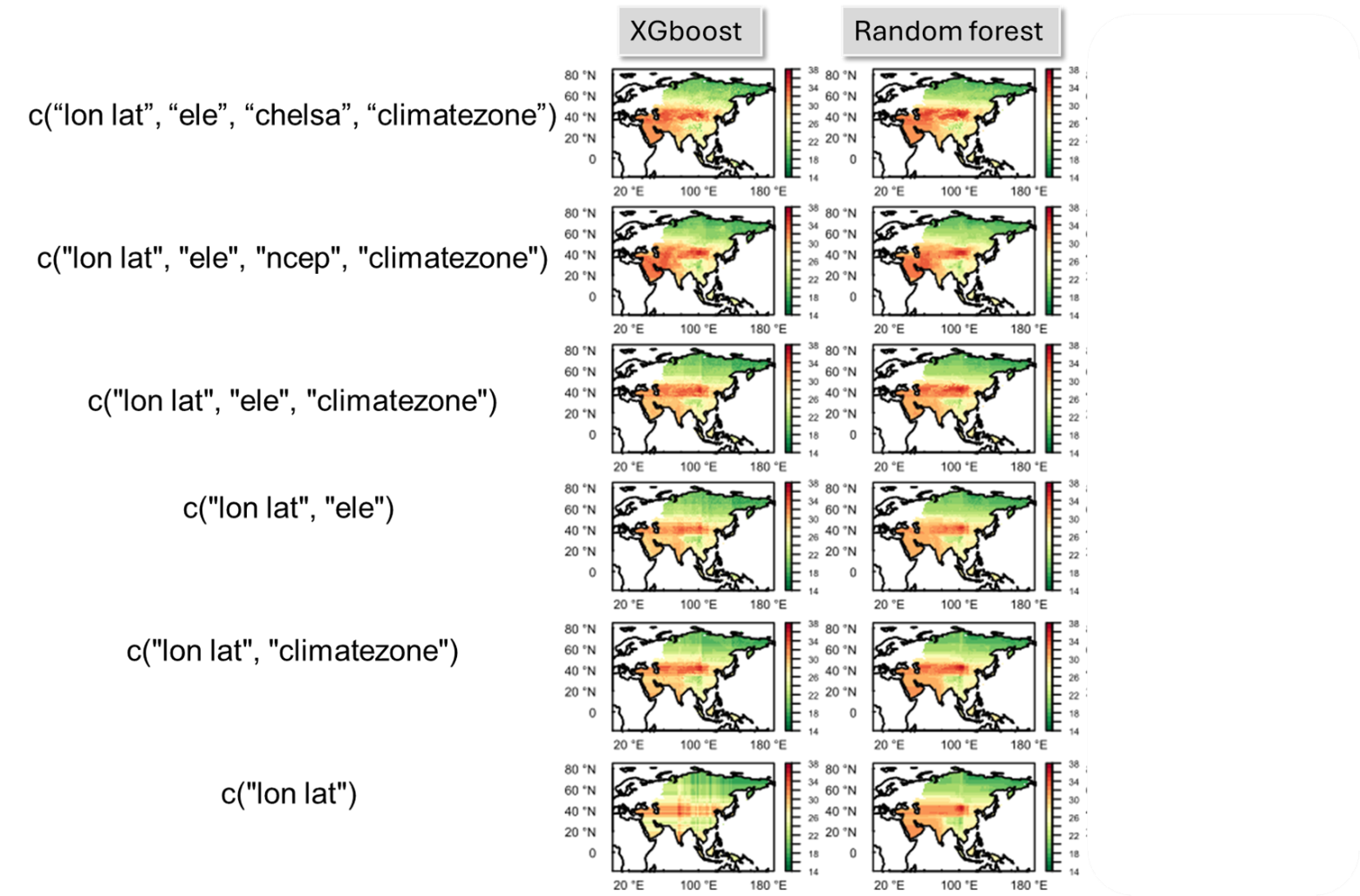


**Fig. S18** Same as Figure 17 but for different parameters combinations.


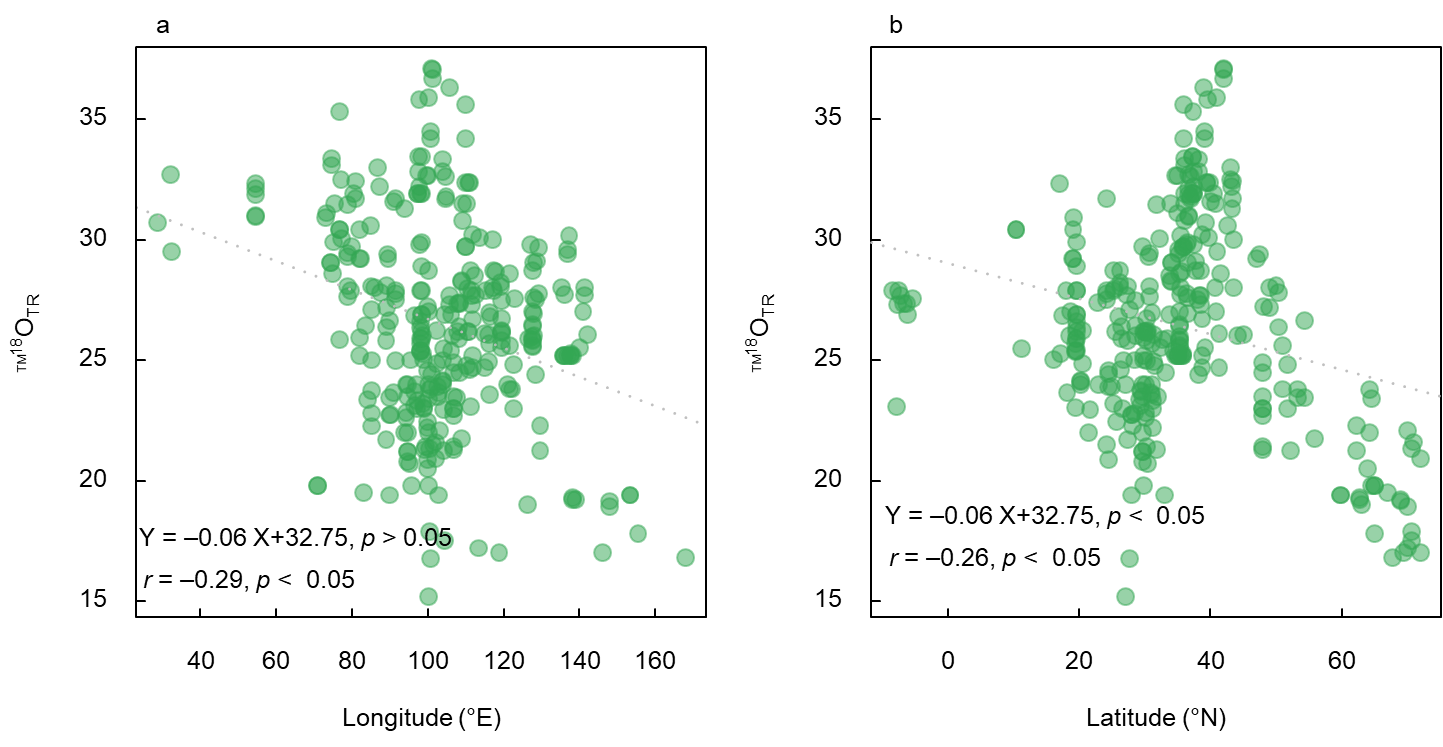


**Fig. S19** The relationship between δ^18^O_TR_ and geographic coordinates. (a) Scatterplot of δ^18^O_TR_ versus Longitude (°E), displaying a non-significant negative correlation. (b) Scatterplot of δ^18^O_TR_ versus Latitude (°N), displaying a significant negative correlation based on linear regression and Pearson’s r statistics. Regression equations and r values are indicated in each panel for reference.


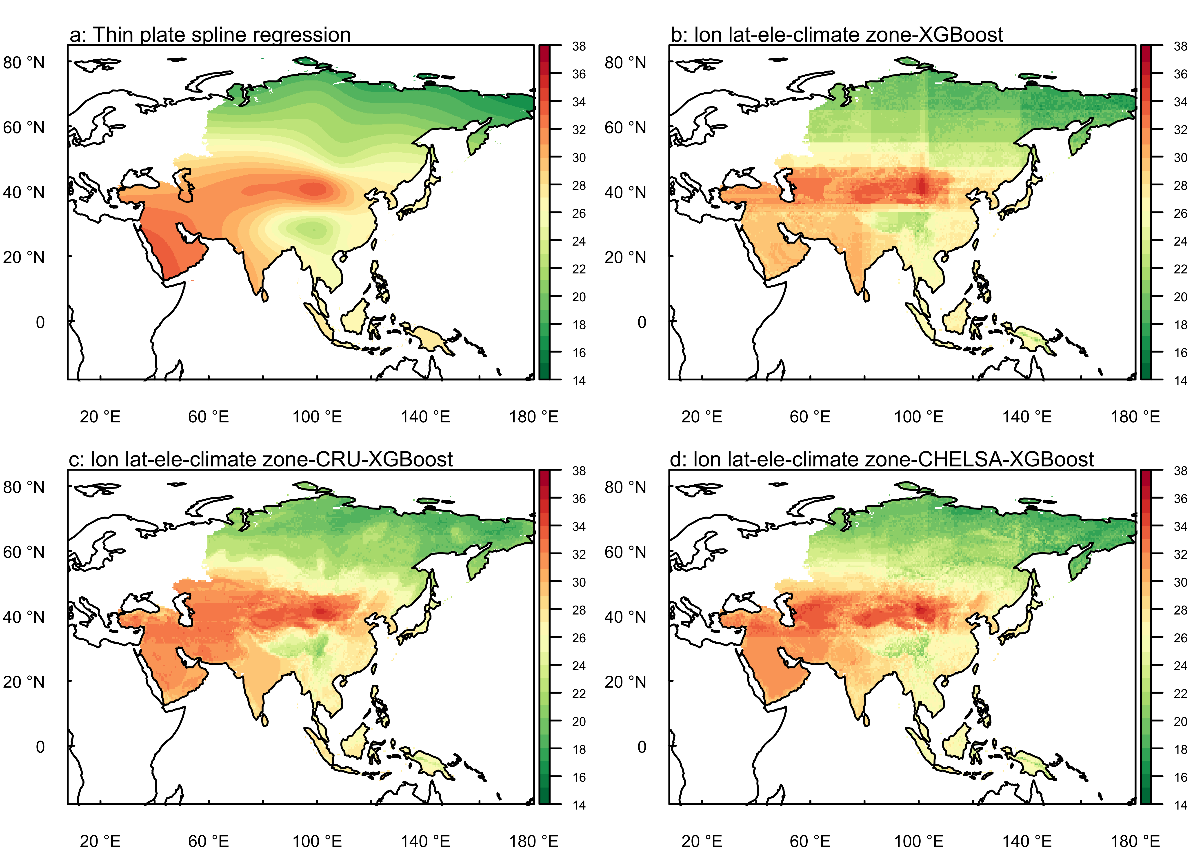


**Fig. S20**. δ¹⁸O_TR_ isoscapes generated using: (a) thin-plate spline regression; (b) XGBoost with longitude, latitude, elevation, and climate zone; (c) XGBoost with longitude, latitude, elevation, climate zone, and CRU predictors (0.5° × 0.5° resolution); (d) XGBoost with longitude, latitude, elevation, climate zone, and CHELSA predictors (30 arc-seconds resolution).

**Table S1** Commonality analysis results showing the unique and shared contributions of CHELSA-based relative humidity (RH) and simulated δ¹⁸O_P_ to the spatial variability of δ¹⁸O_TR_ across Asia.

| Model Name of δ¹⁸O_p_ | Individual effect of RH on δ^18^O_TR_ | Individual effect of δ¹⁸O_p_  on δ^18^O_TR_ | Joint effect on δ^18^O_TR_ | Total effect on δ^18^O_TR_ |
| --- | --- | --- | --- | --- |
| IsoGSM | 18.06% | 36.59% | 3.84% | 58.49% |
| ECHAM5 | 19.46% | 34.43% | 2.44% | 56.33% |
| LMDZ4 | 29.82% | 38.14% | -7.92% | 60.04% |

**Table S2 Data used in the machine learning approaches**

| Data used | Periods | Reference or links |
| --- | --- | --- |
| ETOPO_2022_v1_60s_N90W180 | / | https://www.ncei.noaa.gov/products/etopo-global-relief-model |
| CHELSA | 1981–2010 | [11]  https://envicloud.wsl.ch/#/?bucket=https%3A%2F%2Fos.zhdk.cloud.switch.ch%2Fchelsav2%2F&prefix=%2F |
| CRU TS 4.07 | 1993–2022 | [12]  https://crudata.uea.ac.uk/cru/data/hrg/cru_ts_4.07/ |
| Köppen climate classification | 1986–2010 | [13]  https://koeppen-geiger.vu-wien.ac.at/present.htm |
| NCEP | 1981–2010 | [14]  https://psl.noaa.gov/data/gridded/data.ncep.reanalysis.html |

Note: climate data averaged from May to September are used for the machine learning.

**Table S3** Model Evaluation (XGBoost) using standard accuracy metrics: mean squared error (MSE), root mean squared error (RMSE), mean absolute error (MAE), and coefficient of determination (R²).

| Combinations | MSE | RMSE | MAE | R^2^ | Rank |
| --- | --- | --- | --- | --- | --- |
| Lon lat-ele-chelsa-climatezone | 0.664 | 0.8148 | 0.5651 | 0.9619 | 1 |
| Lon lat-ele-cruvariable-chelsa-ncep-climatezone | 1.0226 | 1.0113 | 0.7736 | 0.9413 | 2 |
| Lon lat-ele-chelsa-ncep-climatezone | 1.1021 | 1.0498 | 0.8029 | 0.9367 | 3 |
| Lon lat-ele-cruvariable-ncep-climatezone | 1.2022 | 1.0964 | 0.8232 | 0.931 | 4 |
| Lon lat-ele-climatezone | 1.2656 | 1.125 | 0.8472 | 0.9274 | 5 |
| lonlat-ele | 1.3025 | 1.1413 | 0.8557 | 0.9252 | 6 |
| Lon lat-ele-ncep-climatezone | 1.3093 | 1.1443 | 0.8759 | 0.9248 | 7 |
| Lon lat-ele-cruvariable-climatezone | 1.3255 | 1.1513 | 0.8631 | 0.9239 | 8 |
| Lon lat-climatezone | 1.4776 | 1.2156 | 0.9157 | 0.9152 | 9 |
| Lon lat | 1.8933 | 1.376 | 1.0443 | 0.8913 | 10 |
| Lon lat-ele-cruvariable-chelsa-climatezone | 2.1543 | 1.4677 | 1.126 | 0.8763 | 11 |

For example, “Lon lat-ele-cruvariable-chelsa-climatezone” stands for the parameters of Longitude, latitude, elevation, CRU variables, CHELSA variables and climate zone, respectively.

**Table S4** Model Evaluation (Random forest) using standard accuracy metrics: mean squared error (MSE), root mean squared error (RMSE), mean absolute error (MAE), and coefficient of determination (R²).

| Combination | MSE | RMSE | MAE | R^2^ | Rank |
| --- | --- | --- | --- | --- | --- |
| Lon lat-ele-chelsa-climatezone | 0.85 | 0.922 | 0.6842 | 0.9512 | 1 |
| Lon lat-ele-cruvariable-chelsa-ncep-climatezone | 0.8817 | 0.939 | 0.7039 | 0.9494 | 2 |
| Lon lat-ele-chelsa-ncep-climatezone | 0.902 | 0.9498 | 0.7196 | 0.9482 | 3 |
| Lon lat-ele-cruvariable-chelsa-climatezone | 0.9236 | 0.961 | 0.7141 | 0.947 | 4 |
| Lon lat-ele | 0.9764 | 0.9881 | 0.7255 | 0.944 | 5 |
| Lon lat-ele-climatezone | 0.9901 | 0.995 | 0.73 | 0.9432 | 6 |
| Lon lat-ele-cruvariable-ncep-climatezone | 1.0029 | 1.0014 | 0.7376 | 0.9424 | 7 |
| Lon lat-climatezone | 1.0108 | 1.0054 | 0.7352 | 0.942 | 8 |
| lonlat-ele-ncep-climatezone | 1.0114 | 1.0057 | 0.7458 | 0.9419 | 9 |
| Lon lat-ele-cruvariable-climatezone | 1.0352 | 1.0175 | 0.7378 | 0.9406 | 10 |
| Lon lat | 1.0581 | 1.0286 | 0.7609 | 0.9393 | 11 |

**References**

1.O'Reilly Sternberg Lda S. Oxygen stable isotope ratios of tree-ring cellulose: the next phase of understanding. *New Phytol* 2009; **181**(3): 553–62.

2.Roden JS, Lin G, Ehleringer JR. A mechanistic model for interpretation of hydrogen and oxygen isotope ratios in tree-ring cellulose. *Geochim. Cosmochim. Acta.* 2000; **64**(1): 21–35.

3.Liu S, Xu C, Fontana C *et al.* Monthly Precipitation Reconstruction in Subtropical South America Using Seasonal Tree-Ring Oxygen Isotopes. *J. Geophys. Res. Biogeosciences* 2023; **128**(12): e2023JG007490.

4.Xu C, Sano M, Yoshimura KEI *et al.* Oxygen isotopes as a valuable tool for measuring annual growth in tropical trees that lack distinct annual rings. *Geochem. J.* 2014; **48**(4): 371–378.

5.R Core Team. R: A language and environment for statistical computing. Vienna, Austria: R Foundation for Statistical Computing; 2025.

6.Chen T, Guestrin C. XGBoost: A scalable tree boosting system. Proc. ACM SIGKDD Int. Conf. Knowl. Discov. Data Min. 2016; 785–794.

7.Breiman L. Random forests. Mach. Learn. 2001; 45(1): 5–32.

8.Cui Y, Tian L, Cai Z *et al.* Spatially inhomogeneous response of precipitation δ^18^O in China to ENSO cycles. *npj clim. Atmos. Sci.* 2025; **8**(1): s41612-025-01057-1

9.Nelson DB, Basler D, Kahmen A. Precipitation isotope time series predictions from machine learning applied in Europe. *Proc. Natl. Acad. Sci. U.S.A.* 2021; **118**(26): e2024107118.

10.Zomer RJ, Xu J, Trabucco A. Version 3 of the global aridity index and potential evapotranspiration database. *Sci. Data*. 2022; **9**(1): 409.

11.Karger DN, Conrad O, Böhner J *et al.* Climatologies at high resolution for the earth’s land surface areas. *Sci. Data*. 2017; **4**(1): 1–20.

12.Harris I, Osborn TJ, Jones P *et al.* Version 4 of the CRU TS monthly high-resolution gridded multivariate climate dataset. *Sci. Data*. 2020; **7**(1): 109.

13.Rubel F, Brugger K, Haslinger K et al. The climate of the European Alps: Shift of very high resolution Köppen-Geiger climate zones 1800–2100. *Meteorol. Z.* 2017; 26(2): 115–125.

14.Kalnay E, Kanamitsu M, Kistler R et al. The NCEP/NCAR 40-Year Reanalysis Project. *Bull. Am. Meteorol. Soc.* 1996; 77(3): 437–472.
